# In-hospital cardiovascular outcomes of COVID-19-associated cytokine storm: a population-level study

**DOI:** 10.3389/fepid.2026.1836720

**Published:** 2026-06-03

**Authors:** Rajveer Sagoo, Navraj S. Sagoo, Paul Murdock, Mohanakrishnan Sathyamoorthy

**Affiliations:** 1 Sathyamoorthy Lab, Department of Internal Medicine, Burnett School of Medicine at Texas, Christian University, Fort Worth, TX, United States; 2 College of Science and Engineering, Texas Christian University, Fort Worth, TX, United States; 3 University of Tennessee-Chattanooga/Erlanger Health System, Chattanooga, TN, United States; 4 Consultants in Cardiovascular Medicine and Science, Fort Worth, TX, United States

**Keywords:** cardiovascular, COVID-19, cytokine storm, mortality, national inpatient sample

## Abstract

**Background:**

In severe COVID-19, hyperinflammatory “Cytokine Storm” (CS) can escalate disease severity, yet large-scale data on its cardiovascular impacts remain limited.

**Objective:**

To assess the influence of CS severity on in-hospital cardiovascular outcomes and mortality in COVID-19 patients.

**Methods:**

A retrospective analysis of the 2021 National Inpatient Sample (NIS) was performed, including adults (≥18 years) hospitalized with a principal COVID-19 diagnosis (ICD-10: U07.1), while excluding other hyperinflammatory conditions. CS was identified and categorized into four severity grades (ICD-10: D89.83). Multivariable logistic regression was utilized to assess associations between CS severity and in-hospital outcomes.

**Results:**

In a weighted cohort of 1,428,892 hospitalized adults with COVID-19, 15,290 (1.1%) had CS. Mortality rose from 8.3% in non-CS patients to 26.9% in grade 4 (adjusted odds ratio [aOR] 3.334; 95% CI [2.810–3.956]), accompanied by increases in cardiac arrest (1.7%–4.4%; aOR 1.742; [1.234–2.459]) and venous thromboembolism (4.8%–9.4%; aOR 1.739; [1.369–2.210]) (*p*_all_ < 0.05). Myocardial Infarction (non-CS: 3.3% vs. Grade 4: 3.1%) and Cardiac Arrhythmias (non-CS: 14.4% vs. grade 4: 14.4%) remained similar across groups but showed decreased adjusted odds in grade 2 (aOR 0.328 [0.221–0.488]; aOR 0.738 [0.641–0.850]) (*p*_all_ < 0.05). Heart failure incidence declined with advancing CS grade (non-CS: 13.5% vs. grade 4: 12.5%), with grades 1 and 2 showing reduced odds (aOR 0.651; [0.475–0.892]), (aOR 0.702; [0.584–0.845]) (*p*_all_ < 0.05). Cardiogenic shock, Ischemic CVA, and Transient Ischemic attack were infrequent and excluded from graded analyses. Compared with non-CS patients, those with grade 4 had a longer median length of stay (5 days vs. 11 days) and higher median total cost (US $44,363 vs. US $142,280) (*p*_all_ < 0.05).

**Conclusion:**

Within this cohort, advanced CS demonstrated an association with increased mortality and select cardiovascular complications in hospitalized COVID-19 patients; Moreover, certain events, such as arrhythmias and heart failure, showed nuanced patterns, underscoring the complex interplay between hyperinflammation and cardiac pathophysiology.

## Introduction

Since its initial identification in December 2019, the novel coronavirus disease (COVID-19) has evolved into the most devastating global public health crises since Influenza-related pandemics in the first half of the twentieth century, resulting in significant morbidity and mortality worldwide. Although the spectrum of COVID-19 illness ranges from asymptomatic infection to critical respiratory failure, a significant subset of hospitalized patients with severe disease demonstrates profound immune dysregulation characterized by unchecked inflammatory pathways, including marked elevations in interleukin-6 (IL-6), tumor necrosis factor-alpha (TNF-α), interleukin-1β (IL-1β), and interferon-γ, a process commonly referred to as a “Cytokine Storm” (CS) (1, 2).

Previous research suggests that CS exacerbates acute lung injury, leading to secondary organ dysfunction, including cardiovascular, renal, and neurologic compromise, and is associated with a higher incidence of thrombotic events, all of which contribute to increased mortality in affected patients (3–5). Yet significant knowledge gaps persist, driven by inconsistent CS definitions arising from a poorly defined pathophysiology, as well as methodological limitations from small, single-center, or region-specific studies (6).

Within this context, this novel study utilizes the 2021 National Inpatient Sample (NIS), the largest publicly available inpatient database in the United States, to deliver a population-level perspective on COVID-19-associated CS. Specifically, we hypothesized that increasing CS severity, as graded by ICD-10 D89.83 sub-classifications, would be associated with a stepwise increase in-hospital mortality and adverse cardiovascular and cerebrovascular outcomes, along with greater healthcare resource utilization. By synthesizing comprehensive, nationally representative data with severity-based analyses, our findings aim to guide more targeted clinical strategies and improve outcomes for hospitalized patients with COVID-19.

## Methods

### Data source and ethics statement

Hospitalization data for this study were obtained from the National Inpatient Sample (NIS) database, a key component of the Healthcare Cost and Utilization Project (HCUP) overseen by the Agency for Healthcare Research and Quality. As the largest publicly available, fully anonymized, all-payer inpatient database in the United States, the NIS compiles billing records from hospitals across the nation and uses validated patient linkage identifiers to track individuals across multiple facilities within each state, while safeguarding privacy through rigorous confidentiality protocols. The database includes both patient and hospital-level information from approximately 4,000 hospitals, representing roughly 20% of all U.S. hospital admissions, more than seven million inpatient stays annually. Through the application of HCUP's discharge-level sampling weights, the NIS can be extrapolated to over 35 million hospitalizations per year, thereby supporting robust national-level analyses. For each patient, up to 40 discharge diagnoses and 25 procedure codes are recorded using the International Classification of Diseases, Tenth Revision (ICD-10).

The NIS contains de-identified patient data and is publicly accessible for research; hence, this study was exempt from Institutional Review Board approval and informed consent requirements. Furthermore, the research was conducted in line with the ethical principles of the 2013 Declaration of Helsinki.

This study adhered to the Strengthening the Reporting of Observational Studies in Epidemiology (STROBE) guidelines, and a completed STROBE checklist can be found in Supplementary Table S2.

### Study population and patient selection

All adult (≥18 years) hospitalizations with a principal diagnosis of COVID-19 in the 2021 NIS were identified by the ICD-10 code U07.1. Admissions in which alternative or concurrent inflammatory processes could confound the diagnosis or clinical evolution of CS were excluded. Specifically, hospitalizations were excluded if they involved sepsis or toxic shock syndrome, chimeric antigen receptor T-cell therapy, graft-vs.-host disease, hemophagocytic lymphohistiocytosis or macrophage activation syndrome, stem cell/bone marrow transplantation, or other immunomodulatory therapies likely to induce a similar hyperinflammatory state. After these exclusions, the remaining sample was stratified based on the presence and severity of CS, as detailed below.

### Defining cytokine storm

Despite considerable discussion in the literature, no universally accepted definition of CS currently exists (7). Investigators have proposed various clinical and laboratory thresholds to capture these hyperinflammatory states, highlighting their inherent complexity and heterogeneity (8). For our analysis, CS was identified using the ICD-10 code D89.83 and its sub-classifications, which encompass the broader nosologic category of “cytokine release syndrome.” A complete listing of these codes is provided in Supplementary Table S1.

Hospitalizations were subsequently categorized into six groups based on these codes: (1) “No CS” (no relevant ICD-10 code); (2) “Grade 1 CS” (D89.831, mild symptoms typically responsive to supportive treatment); (3) “Grade 2 CS” (D89.832, moderate severity requiring intervention such as low-dose vasopressor support or supplemental oxygen); (4) “Grade 3 CS” (D89.833, severe disease requiring advanced supportive care, including high-dose or multiple vasopressors and/or high-flow oxygen); (5) “Grade 4 CS” (D89.834, life-threatening with multi-organ dysfunction necessitating mechanical ventilation or aggressive hemodynamic support); and (6) “Unspecified CS” (D89.839, grade not otherwise specified). This stratification mirrors the clinical-severity framework originally proposed by Lee and colleagues and subsequently formalized in the American Society for Transplantation and Cellular Therapy (ASTCT) consensus grading for cytokine release syndrome (9), in which severity is defined principally by fever, hypotension, hypoxia, and end-organ dysfunction rather than by direct quantification of individual cytokines. Although the ICD-10 framework does not record specific cytokine concentrations, it provides a clinically actionable, internationally standardized stratification suitable for population-level analysis (Supplementary Table S1).

### Data collection

Demographic data (age, sex, primary payer, location, race/ethnicity, and median household income), hospital-level characteristics (census division), and admission profiles (weekend vs. weekday, elective vs. non-elective, emergency) were extracted.

Comorbid conditions were ascertained using Elixhauser comorbidities, a validated determinant of complications and long-term outcomes in numerous populations, including those with COVID-19 (10). Key in-hospital outcomes included mortality, myocardial infarction (MI), heart failure (HF), cardiogenic shock, cardiac arrest, ischemic cerebrovascular accident (CVA), transient ischemic attack (TIA), cardiac arrhythmias (ventricular tachycardia/fibrillation and atrial fibrillation), and venous thromboembolism (VTE) [pulmonary embolism (PE) and deep vein thrombosis (DVT)]. Discharge disposition (e.g., transfer to a skilled nursing facility or home health care) and measures of resource utilization [hospital length of stay (LOS) and hospital charges] were recorded as secondary endpoints. A complete list of ICD-10 diagnosis codes used to define exclusion criteria, comorbidities, and outcomes is provided in Supplementary Table S1.

### Statistical analysis

Data were analyzed using SPSS version 25 (SPSS Inc., Chicago, IL), with appropriate weighting methods recommended by HCUP to generate national estimates (11). Descriptive statistics were generated for demographic, clinical, and hospital variables. Differences in categorical variables between CS severity groups were evaluated using chi-square tests (with Yates' continuity correction or Fisher's exact test, as appropriate), while continuous variables (age, LOS, total charges) were evaluated using the Kruskal–Wallis test if non-normality was detected.

Two multivariable logistic regression models were developed, each adjusted for all demographic, clinical, and hospital characteristics detailed in Table 1, including age, sex, race/ethnicity, primary insurance payer, median household income quartile, hospital census division, weekend vs. weekday admission, elective vs. non-elective admission, emergency department utilization, and the full set of Elixhauser comorbidities. Covariates were selected *a priori* on the basis of clinical relevance and prior literature, and represent conceptually independent domains; no problematic collinearity was identified among the included variables. Model 1 evaluated the association between graded CS severity (Grades 1–4) and in-hospital outcomes, while Model 2 dichotomized the entire cohort into “non-CS” and “CS” (any presence of CS, including unspecified severity grade) to assess the overall impact of CS on clinical endpoints and resource utilization. Adjusted odds ratios (aORs) and 95% confidence intervals (CIs) were estimated, and a *p*-value <0.05 was considered statistically significant.

**Table 1:** Baseline demographics and comorbidities.

Cytokine storm (CS)							
Variable	Non-CS (*n* = 1,413,602)	CS grade 1 (*n* = 1,005)	CS grade 2 (*n* = 2,855)	CS grade 3 (*n* = 1,530)	CS grade 4 (*n* = 800)	CS unspecified (*n* = 9,100)	*P* value
Demographic characteristics							
Age (years)	63 (51–74)	62 (49–73)	58 (47–70)	60 (51–70)	62 (47–71)	60 (48–71)	<0.001
Gender							<0.001
Female	735,553 (52.0%)	515 (51.2%)	1,440 (50.4%)	910 (59.5%)	555 (69.4%)	5,155 (56.6%)	
Male	677,994 (48.0%)	490 (48.8%)	1,415 (49.6%)	620 (40.5%)	245 (30.6%)	3,945 (43.4%)	
Race							
White	887,188 (62.8%)	780 (77.6%)	1,845 (64.6%)	965 (63.1%)	505 (63.1%)	4,975 (54.7%)	
Black	210,105 (14.9%)	75 (7.5%)	295 (10.3%)	210 (13.7%)	75 (9.4%)	1,665 (18.3%)	
Hispanic	193,365 (13.7%)	105 (10.4%)	495 (17.3%)	230 (15.0%)	170 (21.3%)	1,580 (17.4%)	
Asian or Pacific Islander	33,810 (2.4%)	20 (2.0%)	55 (1.9%)	15 (1.0%)	10 (1.3%)	230 (2.5%)	
Native American	10,760 (0.8%)	0 (0.0%)	5 (0.2%)	0 (0.0%)	0 (0.0%)	35 (0.4%)	
Other	39,940 (2.8%)	20 (2.0%)	115 (4.0%)	80 (5.2%)	35 (4.4%)	320 (3.5%)	
Primary payer							<0.001
Medicare	655,783 (46.4%)	470 (47.3%)	1,040 (36.4%)	620 (40.5%)	315 (39.4%)	3,515 (38.6%)	
Medicaid	173,830 (12.3%)	145 (14.4%)	375 (13.1%)	130 (8.5%)	115 (14.4%)	1,115 (12.3%)	
Private Insurance	461,144 (32.6%)	330 (32.8%)	1,070 (37.5%)	590 (38.6%)	260 (32.5%)	3,435 (37.7%)	
Other	51,500 (3.6%)	35 (3.5%)	150 (5.3%)	65 (4.2%)	40 (5.0%)	405 (4.5%)	
Median household income							<0.001
$1–55,999	447,503 (31.7%)	365 (36.3%)	820 (28.7%)	480 (31.4%)	210 (26.3%)	2,940 (32.3%)	
$56,000–70,999	385,879 (27.3%)	315 (31.3%)	750 (26.3%)	460 (30.1%)	240 (30.0%)	2,160 (23.7%)	
$71,000–93,999	328,194 (23.2%)	215 (21.4%)	770 (27.0%)	345 (22.5%)	220 (27.5%)	2,095 (23.0%)	
$94,000+	230,080 (16.3%)	75 (7.5%)	470 (16.5%)	225 (14.7%)	120 (15.0%)	1,755 (19.3%)	
Patient location							<0.001
Central counties of metro areas of ≥1 million population	360,015 (25.5%)	250 (24.9%)	1,300 (45.5%)	685 (44.8%)	275 (34.4%)	3,005 (33.0%)	
Fringe counties of metro areas of ≥1 million population	341,649 (24.2%)	170 (16.9%)	325 (11.4%)	110 (7.2%)	180 (22.5%)	2,500 (27.5%)	
Counties in metro areas of 250,000–999,999 population	290,230 (20.5%)	205 (20.4%)	470 (16.5%)	250 (16.3%)	95 (11.9%)	1,770 (19.5%)	
Counties in metro areas of 50,000–249,999 population	142,985 (10.1%)	90 (9.0%)	275 (9.6%)	265 (17.3%)	125 (15.6%)	490 (5.4%)	
Micropolitan counties	150,804 (10.6%)	175 (17.4%)	325 (11.4%)	80 (5.2%)	85 (10.6%)	765 (8.4%)	
Not metropolitan or micropolitan counties	122,869 (8.7%)	115 (11.4%)	155 (5.4%)	135 (8.8%)	40 (5.0%)	555 (6.1%)	
Clinical characteristics							
Cardiovascular							
Hypertension, uncomplicated	565,639 (40.0%)	460 (45.8%)	1,120 (39.2%)	540 (35.3%)	300 (37.5%)	3,785 (41.6%)	<0.001
Hypertension, complicated	0	160 (15.9%)	480 (16.8%)	285 (18.6%)	165 (20.6%)	1,700 (18.7%)	<0.001
Peripheral vascular disease	51,520 (3.6%)	40 (4.0%)	20 (0.7%)	65 (4.2%)	30 (3.8%)	220 (2.4%)	<0.001
Pulmonary circulation disorder	36,545 (2.6%)	15 (1.5%)	60 (2.1%)	50 (3.3%)	15 (1.9%)	205 (2.3%)	<0.001
Valve disease	54,130 (3.8%)	50 (5.0%)	30 (1.1%)	50 (3.3%)	40 (5.0%)	310 (3.4%)	<0.001
Neurological							
Cerebrovascular disease	19,750 (1.4%)	15 (1.5%)	45 (1.6%)	20 (1.3%)	35 (4.4%)	145 (1.6%)	<0.001
Cerebrovascular sequelae	21,415 (1.5%)	5 (0.5%)	20 (0.7%)	15 (1.0%)	5 (0.6%)	85 (0.9%)	<0.001
Dementia	91,785 (6.5%)	75 (7.5%)	145 (5.1%)	75 (4.9%)	20 (2.5%)	350 (3.8%)	<0.001
Neurological movement disorder	31,760 (2.2%)	40 (4.0%)	50 (1.8%)	5 (0.3%)	20 (2.5%)	100 (1.1%)	<0.001
Other neurological disorders	124,355 (8.8%)	55 (5.5%)	150 (5.3%)	140 (9.2%)	120 (15.0%)	865 (9.5%)	<0.001
Neurological seizure disorder	35,575 (2.5%)	10 (1.0%)	25 (0.9%)	30 (2.0%)	15 (1.9%)	175 (1.9%)	<0.001
Paralysis	29,375 (2.1%)	20 (2.0%)	25 (0.9%)	25 (1.6%)	35 (4.4%)	170 (1.9%)	<0.001
Respiratory							
Chronic lung disease	324,324 (22.9%)	210 (20.9%)	560 (19.6%)	315 (20.6%)	125 (15.6%)	1,670 (18.4%)	<0.001
Renal							
Renal failure, moderate	156,060 (11.0%)	120 (11.9%)	285 (10.0%)	115 (7.5%)	85 (10.6%)	805 (8.8%)	<0.001
Renal failure, severe	68,020 (4.8%)	25 (2.5%)	90 (3.2%)	55 (3.6%)	20 (2.5%)	340 (3.7%)	<0.001
Liver/GI							
Liver disease, mild	69,365 (4.9%)	40 (4.0%)	175 (6.1%)	60 (3.9%)	70 (8.8%)	505 (5.5%)	<0.001
Liver disease, severe	7,815 (0.6%)	10 (1.0%)	0 (0.0%)	5 (0.3%)	10 (1.3%)	40 (0.4%)	<0.001
Peptic ulcer	5,785 (0.4%)	0 (0.0%)	15 (0.5%)	30 (2.0%)	0 (0.0%)	30 (0.3%)	<0.001
Endocrine							
Diabetes, complicated	329,859 (23.3%)	230 (22.9%)	680 (23.8%)	430 (28.1%)	240 (30.0%)	2,355 (25.9%)	<0.001
Diabetes, uncomplicated	183,704 (13.0%)	75 (7.5%)	210 (7.4%)	110 (7.2%)	85 (10.6%)	990 (10.9%)	<0.001
Hypothyroidism	187,535 (13.3%)	175 (17.4%)	390 (13.7%)	120 (7.8%)	85 (10.6%)	1,005 (11.0%)	<0.001
Psychiatric/Substance use							
Depression	153,505 (10.9%)	120 (11.9%)	245 (8.6%)	105 (6.9%)	65 (8.1%)	800 (8.8%)	<0.001
Psychoses	46,915 (3.3%)	50 (5.0%)	70 (2.5%)	40 (2.6%)	20 (2.5%)	185 (2.0%)	<0.001
Alcohol abuse	25,280 (1.8%)	15 (1.5%)	30 (1.1%)	35 (2.3%)	15 (1.9%)	185 (2.0%)	<0.001
Drug abuse	24,980 (1.8%)	25 (2.5%)	45 (1.6%)	40 (2.6%)	15 (1.9%)	110 (1.2%)	<0.001
Cancer							
Metastatic cancer	11,460 (0.8%)	5 (0.5%)	5 (0.2%)	10 (0.7%)	10 (1.3%)	40 (0.4%)	<0.001
Solid cancer	30,675 (2.2%)	10 (1.0%)	25 (0.9%)	15 (1.0%)	10 (1.3%)	130 (1.4%)	<0.001
Hematologic							
Anemia	192,470 (13.6%)	105 (10.4%)	345 (12.1%)	205 (13.4%)	190 (23.8%)	1,370 (15.1%)	<0.001
Coagulopathy	141,800 (10.0%)	125 (12.4%)	300 (10.5%)	180 (11.8%)	165 (20.6%)	1,455 (16.0%)	<0.001
Autoimmune							
Autoimmune disease	48,880 (3.5%)	55 (5.5%)	115 (4.0%)	80 (5.2%)	35 (4.4%)	290 (3.2%)	<0.001
AIDS	5,935 (0.4%)	5 (0.5%)	10 (0.4%)	10 (0.7%)	5 (0.6%)	35 (0.4%)	0.245
Metabolic/Other							
Obesity	452,949 (32.0%)	380 (37.8%)	1,260 (44.1%)	650 (42.5%)	410 (51.2%)	3,945 (43.4%)	<0.001
Weight loss (cachexia)	75,735 (5.4%)	45 (4.5%)	140 (4.9%)	115 (7.5%)	90 (11.3%)	530 (5.8%)	<0.001
Hospital characteristics & admission profiles							
Census division							<0.001
New England	48,680 (3.4%)	0 (0.0%)	0 (0.0%)	5 (0.3%)	0 (0.0%)	120 (1.3%)	
Middle Atlantic	178,630 (12.6%)	15 (1.5%)	20 (0.7%)	40 (2.6%)	10 (1.3%)	845 (9.3%)	
East North Central	227,850 (16.1%)	115 (11.4%)	95 (3.3%)	70 (4.6%)	115 (14.4%)	1,960 (21.5%)	
West North Central	87,135 (6.2%)	0 (0.0%)	10 (0.4%)	30 (2.0%)	15 (1.9%)	250 (2.7%)	
South Atlantic	327,963 (23.2%)	195 (19.4%)	860 (30.1%)	655 (42.8%)	195 (24.4%)	1,780 (19.6%)	
East South Central	98,885 (7.0%)	590 (58.7%)	1,030 (36.1%)	310 (20.3%)	135 (16.9%)	1,260 (13.8%)	
West South Central	193,450 (13.7%)	55 (5.5%)	480 (16.8%)	165 (10.8%)	135 (16.9%)	1,520 (16.7%)	
Mountain	103,989 (7.4%)	5 (0.5%)	10 (0.4%)	40 (2.6%)	10 (1.3%)	200 (2.2%)	
Pacific	147,019 (10.4%)	30 (3.0%)	350 (12.3%)	215 (14.1%)	185 (23.1%)	1,165 (12.8%)	
Emergency department							<0.001
No ED record	154,719 (10.9%)	40 (4.0%)	115 (4.0%)	140 (9.2%)	60 (7.5%)	770 (8.5%)	
Emergency department revenue code on record	896,463 (63.4%)	670 (66.7%)	1,695 (59.4%)	765 (50.0%)	460 (57.5%)	4,785 (52.6%)	
Positive emergency department charge (when revenue center codes are not available)	357,784 (25.3%)	295 (29.4%)	1,045 (36.6%)	625 (40.8%)	280 (35.0%)	3,535 (38.8%)	
Emergency department CPT procedure code on record	10 (0.0%)	0 (0.0%)	0 (0.0%)	0 (0.0%)	0 (0.0%)	0 (0.0%)	
Condition code P7 indication of ED admission, point of origin of ED, or admission source of ED	4,625 (0.3%)	0 (0.0%)	0 (0.0%)	0 (0.0%)	0 (0.0%)	10 (0.1%)	
Weekend vs. weekday admission							<0.001
Weekday	1,042,982 (73.8%)	745 (74.1%)	2,035 (71.3%)	1,160 (75.8%)	565 (70.6%)	6,740 (74.1%)	
Weekend	370,614 (26.2%)	260 (25.9%)	820 (28.7%)	370 (24.2%)	235 (29.4%)	2,360 (25.9%)	
Elective vs. non-elective admission							<0.001
Non-elective	1,376,792 (97.4%)	1,000 (99.5%)	2,820 (98.8%)	1,505 (98.4%)	800 (100.0%)	8,935 (98.2%)	
Elective	35,595 (2.5%)	5 (0.5%)	25 (0.9%)	25 (1.6%)	0 (0.0%)	160 (1.8%)	

Age is reported as median (IQR). IQR, interquartile range.

## Results

### Patient population and selection

**Figure 1:**
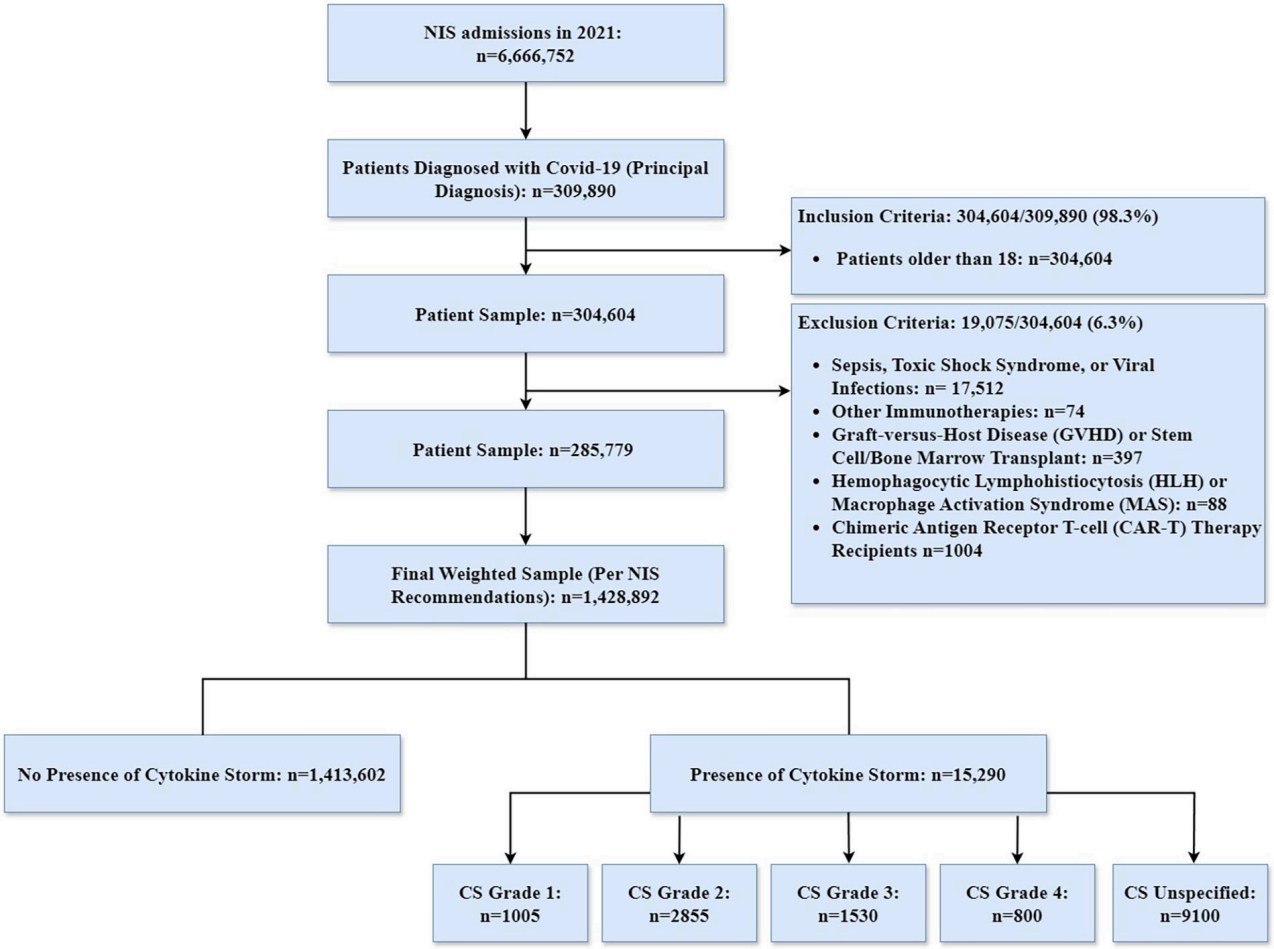
Flowchart of patient selection. Flowchart detailing the screening of 6,666,752 hospitalizations from the 2021 National Inpatient Sample (NIS) for a principal diagnosis of COVID-19. Patients under 18 years of age and those with other hyperinflammatory conditions were excluded, resulting in a final weighted sample of 1,428,892 hospitalizations. Among these, 15,290 had a documented diagnosis of Cytokine Storm (CS), further stratified into CS grades 1, 2, 3, 4, or unspecified. CAR T, chimeric antigen receptor T-cell therapy; CS, cytokine storm; GvHD, graft-vs.-host disease; HLH, hemophagocytic lymphohistiocytosis; MAS, macrophage activation syndrome; NIS, national inpatient sample.

A total of 1,413,602 weighted hospitalizations with a principal diagnosis of COVID-19 were identified from the 2021 NIS after applying inclusion and exclusion criteria (Figure 1). Among these, 15,290 (1.1%) patients had a diagnosis of CS. Within this CS cohort, the distribution of severity grades was as follows: Grade 1 (1,005 patients; 6.6%), Grade 2 (2,855 patients; 18.7%), Grade 3 (1,530 patients; 10.0%), Grade 4 (800 patients; 5.2%), and unspecified grade (9,100 patients; 59.5%). The remaining 1,413,602 (98.9%) COVID-19 admissions did not exhibit CS. All subsequent analyses reflect this stratification of the study population.

### Baseline demographics and comorbidities

Table 1 summarizes demographic and clinical characteristics. The median age was similar across groups but was lowest in Grade 2 (58 years [IQR 47–70]) and highest in the non-CS group (63 years [51–74]) (*p* < 0.001). Female patients constituted 52% of non-CS hospitalizations, but this proportion increased in Grade 3 (59.5%) and Grade 4 (69.4%) CS (*p* < 0.001). Racial distribution varied significantly (*p* < 0.001). White patients predominated in all groups (Range: 54.7%–77.6%), whereas Hispanic patients were more prevalent in Grade 3 (15.0%) and Grade 4 (21.3%) than in the non-CS cohort (13.7%).

Insurance coverage demonstrated significant variation (*p* < 0.001), with Medicare and private insurance being the most frequent payment sources. Although significance was observed for median household income (*p* < 0.001), no notable trend by CS grade emerged.

Across the study population, the presence and severity of CS was associated with distinct comorbidity patterns. Cardiovascular conditions, particularly uncomplicated and complicated hypertension, were frequent in all cohorts and remained broadly similar to non-CS levels (*p*_all_ < 0.001). Neurological conditions were less common overall, although cerebrovascular disease was notably higher in Grade 4 (4.4%) compared to the non-CS cohort (1.4%) (*p* < 0.001).

Chronic lung disease exhibited a progressive decline across cohorts, decreasing from 22.9% in the non-CS group to 15.6% in Grade 4 (*p* < 0.001). Moreover, the prevalence of moderate renal failure remained largely comparable across both non-CS and CS cohorts, whereas severe renal failure generally remained infrequent (*p*_all_ < 0.001).

Among endocrine disorders, complicated diabetes increased across CS grades (non-CS: 23.3%, Grade 4: 30.0%) (*p* < 0.001). Obesity was also considerably more frequent in CS cohorts (Range: 37.8%–51.2%) than in non-CS (32.0%) (*p* < 0.001). Psychosocial comorbidities, including depression and substance use, were present across all groups, albeit with modest differences (*p* < 0.001). Notably, coagulopathy and anemia both increased significantly with advancing CS severity, from 10.0% and 13.6% in the non-CS group to 20.6% and 23.8%, respectively, in Grade 4 (*p* < 0.001).

### Hospital characteristics and admission profiles

Regionally, the South Atlantic and East South-Central divisions accounted for a substantial proportion of non-CS admissions (*p* < 0.001). Elective admissions were consistently rare across all groups, with nearly all patients admitted non-electively (Range: 97.4%–100.0%; *p* < 0.001). Moreover, weekday admissions predominated (Range: 70.6%–75.8%; *p* < 0.001), and emergency department utilization remained high (Range: 50.0%–66.7%; *p* < 0.001) across all cohorts (Table 1).

### Graded analysis of in-hospital outcomes

Table 2 summarizes the in-hospital event rates and resource utilization stratified by CS severity. Mortality increased significantly from 8.3% among patients without CS to 26.9% in Grade 4 CS (*p* < 0.001). Cardiac complications similarly rose, with cardiogenic shock and cardiac arrest reaching 1.3% and 4.4%, respectively, in Grade 4, compared with 0.3% and 1.7% in those without CS (*p*_all_ < 0.001). HF and MI remained frequent, with small numeric differences between non-CS and Grade 4 cohorts (*p*_all_ < 0.001). Cardiac arrhythmias were frequent across all severity groups, with comparable rates in patients without CS (14.4%) and those with Grade 4 CS (14.4%). Specifically, the prevalence of atrial fibrillation was similar (13.3% vs. 13.1%), and ventricular tachycardia/fibrillation increased only modestly (1.6% vs. 1.9%) (*p*_all_ < 0.001). In contrast, VTE increased to 9.4% in Grade 4 CS compared with 4.8% in the non-CS group, driven by increases in both PE (5.6% vs. 3.7%) and DVT (3.8% vs. 1.6%) (*p*_all_ < 0.001). Cerebrovascular events were rare but varied significantly: TIAs were more common in Grade 1–2 but absent in Grade 3–4, while Ischemic CVA peaked at 0.6% in Grade 4 (*p* < 0.001).

**Table 2:** Graded analysis of in-hospital event rates.

Cytokine storm (CS)							
Variable	Non-CS (*n* = 1,413,602)	CS grade 1 (*n* = 1,005)	CS grade 2 (*n* = 2,855)	CS grade 3 (*n* = 1,530)	CS grade 4 (*n* = 800)	CS unspecified (*n* = 9,100)	*P* value
Clinical outcomes							
Mortality	116,910 (8.3%)	20 (2.0%)	85 (3.0%)	185 (12.1%)	215 (26.9%)	1,235 (13.6%)	<0.001
Cardiogenic shock	3,860 (0.3%)	0 (0.0%)	5 (0.2%)	0 (0.0%)	10 (1.3%)	45 (0.5%)	<0.001
Cardiac arrhythmias	203,120 (14.4%)	125 (12.4%)	240 (8.4%)	195 (12.7%)	115 (14.4%)	1,075 (11.8%)	<0.001
Atrial fibrillation	188,315 (13.3%)	125 (12.4%)	230 (8.1%)	180 (11.8%)	105 (13.1%)	930 (10.2%)	<0.001
Ventricular Tachycardia/Fibrillation	22,360 (1.6%)	0 (0.0%)	15 (0.5%)	25 (1.6%)	15 (1.9%)	180 (2.0%)	<0.001
Cardiac arrest	23,325 (1.7%)	0 (0.0%)	10 (0.4%)	25 (1.6%)	35 (4.4%)	360 (4.0%)	<0.001
Heart failure	190,745 (13.5%)	80 (8.0%)	225 (7.9%)	195 (12.7%)	100 (12.5%)	870 (9.6%)	<0.001
Myocardial infarction	46,190 (3.3%)	25 (2.5%)	25 (0.9%)	45 (2.9%)	25 (3.1%)	330 (3.6%)	<0.001
Venous thromboembolism	67,605 (4.8%)	35 (3.5%)	120 (4.2%)	125 (8.2%)	75 (9.4%)	585 (6.4%)	<0.001
Pulmonary embolism	52,280 (3.7%)	30 (3.0%)	100 (3.5%)	55 (3.6%)	45 (5.6%)	385 (4.2%)	0.004
Deep vein thrombosis	22,615 (1.6%)	10 (1.0%)	25 (0.9%)	80 (5.2%)	30 (3.8%)	275 (3.0%)	<0.001
Transient ischemic attack	1,700 (0.1%)	10 (1.0%)	10 (0.4%)	0 (0.0%)	0 (0.0%)	20 (0.2%)	<0.001
Ischemic CVA	6,645 (0.5%)	5 (0.5%)	0 (0.0%)	5 (0.3%)	5 (0.6%)	65 (0.7%)	<0.001
Discharge disposition							<0.001
Routine	849,633 (60.1%)	690 (68.7%)	2,115 (74.1%)	815 (53.3%)	285 (35.6%)	5,205 (57.2%)	
Transfer to short-term hospital	35,335 (2.5%)	0 (0.0%)	25 (0.9%)	60 (3.9%)	50 (6.3%)	230 (2.5%)	
Skilled nursing facility	174,830 (12.4%)	120 (11.9%)	180 (6.3%)	200 (13.1%)	130 (16.3%)	1,125 (12.4%)	
Other transfers	214,485 (15.2%)	160 (15.9%)	435 (15.2%)	265 (17.3%)	105 (13.1%)	1,195 (13.1%)	
Against medical advice	21,745 (1.5%)	15 (1.5%)	15 (0.5%)	5 (0.3%)	15 (1.9%)	100 (1.1%)	
Resource utilization							
Length of stay (days)	5 (3–9)	5 (3–7)	4 (3–7)	9 (6–15)	11 (7–21)	7 (5–13)	<0.001
Total charges ($)	44,363 (25,546–79,285)	49,814 (30,784–70,859)	53,379 (33,770–77,664)	91,344 (51,932–149,673)	142,280 (77,244–235,185)	76,335 (45,847–141,417)	<0.001

Length of stay and total charges are reported as median (IQR). IQR, interquartile range.

The severity of CS likewise influenced discharge disposition, with a lower percentage of routine discharges in grades 3 and 4 (53.3% and 35.6%, respectively) than among those without CS (60.1%). Resource utilization metrics further underscored this trend (*p* < 0.001): median LOS increased from 5 days in the non-CS group to 11 days for grade 4 CS, while total charges nearly tripled among patients with grade 4 CS compared with no CS (median $142,280 vs. $44,363).

**Figure 2:**
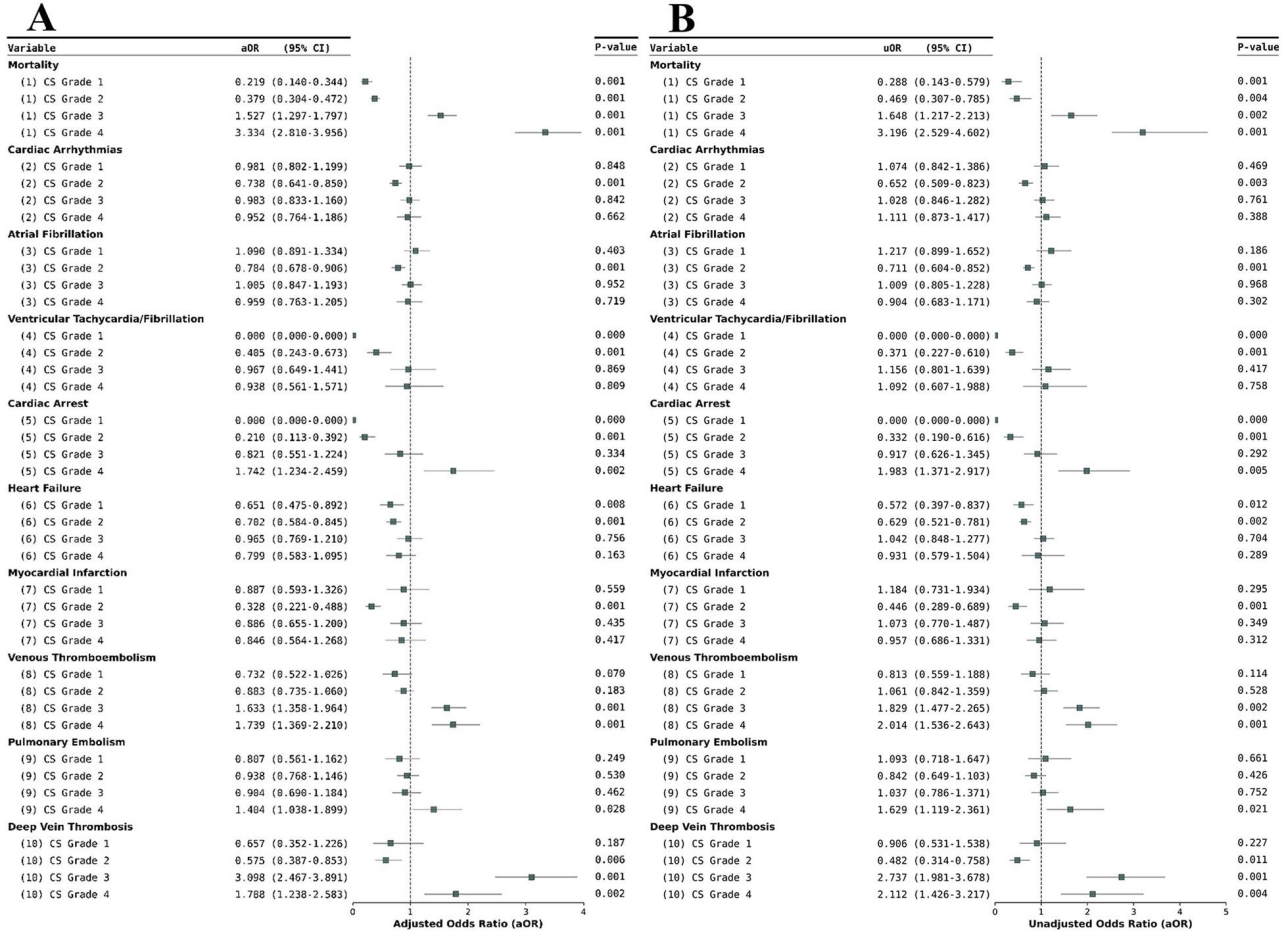
Graded analysis of clinical outcomes by CS severity. Forest plots (Panel **A**, adjusted; Panel **B**, unadjusted) displaying odds ratios (ORs) and 95% confidence intervals for in-hospital outcomes, stratified by CS severity (grades 1–4). Each outcome is presented separately to compare the odds among patients in each CS grade to those without CS. Cardiogenic shock, ischemic cerebrovascular accident (CVA), and transient ischemic attack (TIA) were excluded due to low event rates in each grade. aOR, adjusted odds ratio; CI, confidence interval; CS, cytokine storm; OR, odds ratio; uOR, unadjusted odds ratio.

After multivariable adjustment, a nuanced pattern of associations emerged across CS severity, as shown in Figure 2. Compared with no CS, Grades 1 and 2 were associated with significantly lower in-hospital mortality (adjusted odds ratio [aOR] 0.219 [0.140–0.344] and 0.379 [0.304–0.472], respectively; both *p* < 0.001), whereas odds rose markedly in Grades 3 and 4 (aOR 1.527 [1.297–1.797] and 3.334 [2.810–3.956]; both *p* < 0.001). This trend was mirrored for cardiac arrest, with substantially lower odds in early grades but a pronounced increase at Grade 4 (aOR 1.742 [1.234–2.459]; *p* = 0.002). Although arrhythmic complications and MI showed consistently reduced odds in Grade 2, significant differences were generally not observed. HF demonstrated lower odds in Grades 1 (aOR 0.651 [0.475–0.892]; *p* = 0.008) and 2 (aOR 0.702 [0.584–0.845]; *p* < 0.001) but converged to no difference in Grades 3 and 4. In contrast, odds of venous thromboembolism increased steeply in Grades 3 and 4 (aOR 1.633 [1.358–1.964] and 1.739 [1.369–2.210], respectively; both *p* < 0.001), driven by higher odds of both PE (Grade 4: 1.404 [1.038–1.899]; *p* = 0.028) and DVT (Grade 4: 1.788 [1.238–2.583]; *p* = 0.002). Given their low event counts, cardiogenic shock, TIA, and Ischemic CVA were excluded from the graded analyses.

### Dichotomized analysis of in-hospital outcomes

**Figure 3:**
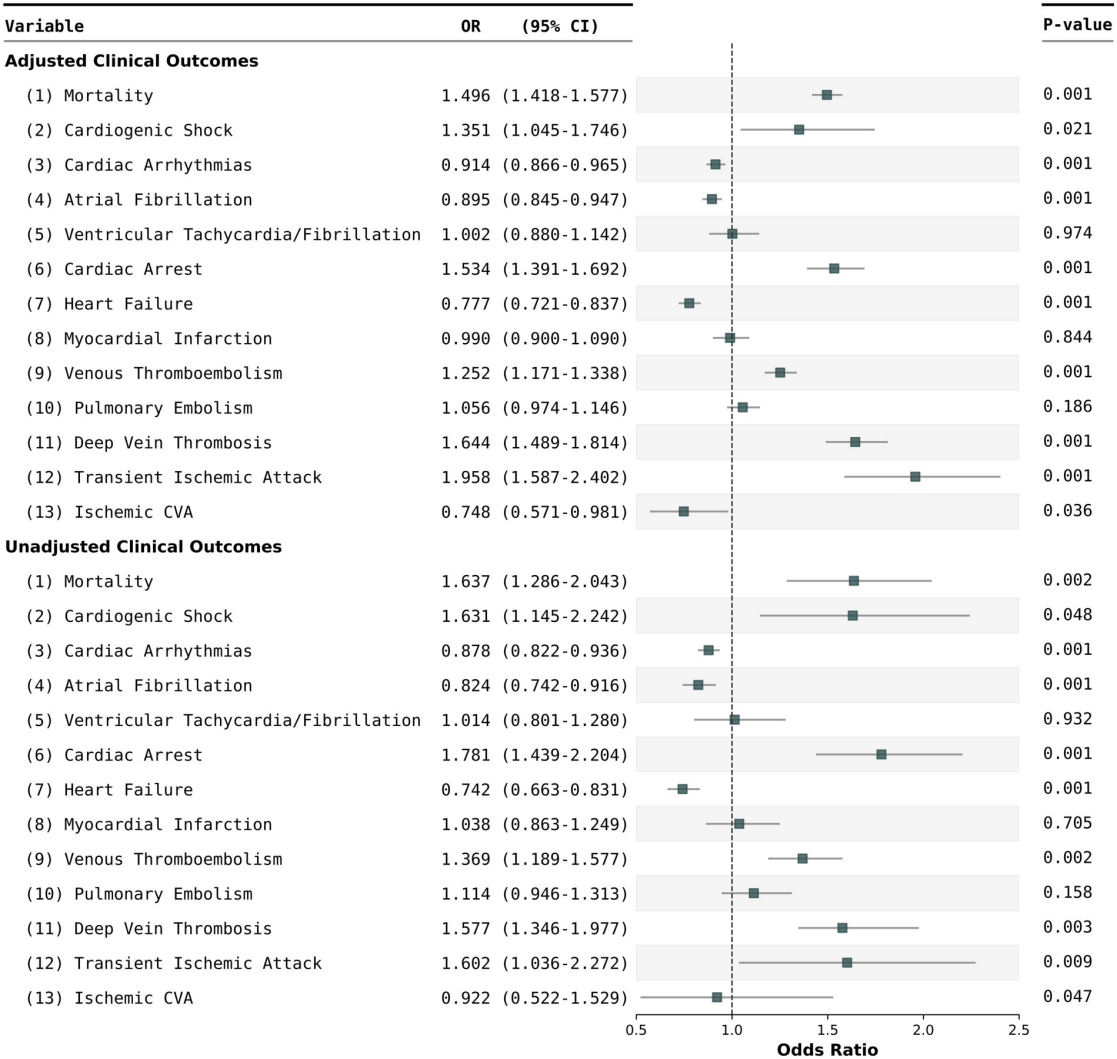
Dichotomized analysis of clinical outcomes by presence of CS forest plots showing both adjusted and unadjusted ORs (with 95% confidence intervals) for key in-hospital outcomes, comparing patients with vs. without CS in the context of COVID-19. aOR, adjusted odds ratio; CI, confidence interval; CS, cytokine storm; CVA, cerebrovascular accident; OR, odds ratio. *Central Illustration: Cardiovascular Outcomes of COVID-19-Associated Cytokine Storm.* COVID-19, coronavirus disease 2019; ; HF, heart failure; LOS, length of stay; MI, myocardial infarction; NIS, national inpatient sample; TIA, transient ischemic attack; VTE, venous thromboembolism.

Table 3 summarizes in-hospital event rates and resource utilization stratified by the presence or absence of CS, while Figure 3 presents the corresponding odds ratios. Mortality was significantly higher in the CS cohort vs. non-CS (11.4% vs. 8.3%; *p* < 0.001), which paralleled the increased adjusted odds observed (aOR: 1.496 [1.418–1.577]; *p* < 0.001). Similarly, rates of cardiac arrest were greater among patients with CS (2.8% vs. 1.7%; *p* < 0.001), and this difference persisted after multivariable adjustment (aOR:  1.534 [1.391–1.692]; *p* < 0.001). In contrast, cardiac arrhythmias occurred less frequently in the CS group (11.4% vs. 14.4%; *p* < 0.001), with corresponding reduced odds (aOR: 0.914 [0.866–0.965]; *p* < 0.001), driven in part by atrial fibrillation (OR 0.895 [0.845–0.947]; *p* < 0.001). Ventricular tachycardia/fibrillation was similar between cohorts (1.5% vs. 1.6%; *p* < 0.659), with no significant difference observed in odds (aOR: 1.002 [0.880–1.142]; *p* < 0.974). Although cardiogenic shock remained infrequent in both groups (0.4% vs. 0.3%; *p* < 0.006), it was significantly elevated in adjusted odds (aOR: 1.351 [1.045–1.746]; *p* < 0.021).

**Table 3:** Dichotomized analysis of in-hospital event rates.

Cytokine storm (CS)			
Variable	Non-CS (*n* = 1,413,602)	CS (*n* = 15,290)	*P* value
Clinical outcomes			
Mortality	116,910 (8.3%)	1,740 (11.4%)	<0.001
Cardiogenic shock	3,860 (0.3%)	60 (0.4%)	0.006
Cardiac arrhythmias	203,120 (14.4%)	1,750 (11.4%)	<0.001
Atrial fibrillation	188,315 (13.3%)	1,570 (10.3%)	<0.001
Ventricular Tachy/Fibrillation	22,360 (1.6%)	235 (1.5%)	0.659
Cardiac arrest	23,325 (1.7%)	430 (2.8%)	<0.001
Heart failure	190,745 (13.5%)	1,470 (9.6%)	<0.001
Myocardial infarction	46,190 (3.3%)	450 (2.9%)	0.025
Venous thromboembolism	67,605 (4.8%)	940 (6.1%)	<0.001
Pulmonary embolism	52,280 (3.7%)	615 (4.0%)	0.035
Deep vein thrombosis	22,615 (1.6%)	420 (2.7%)	<0.001
Transient ischemic attack	1,700 (0.1%)	40 (0.3%)	<0.001
Ischemic CVA	6,645 (0.5%)	80 (0.5%)	0.340
Patient disposition			<0.001
Routine	849,633 (60.1%)	9,110 (59.6%)	
Short-term hospital	35,335 (2.5%)	365 (2.4%)	
Skilled nursing facility	174,830 (12.4%)	1,755 (11.5%)	
Home health care	214,485 (15.2%)	2,160 (14.1%)	
Against medical advice	21,745 (1.5%)	150 (1.0%)	
Resource utilization			
Length of stay (days)	5 (3–9)	7 (4–12)	0.009
Total charges ($)	$44,596 (25,549–79,299)	$69,713 (42,873–129,367)	<0.001

Length of stay and total charges are reported as median (IQR). IQR, interquartile range.

HF was notably reduced in the CS cohort vs. non-CS (9.6% vs. 13.5%; *p* < 0.001), and this association remained significant in adjusted analyses (aOR: 0.777 [0.721–0.837]; *p* < 0.001), which was surprising. Although the incidence of MI appeared modestly lower in the CS group (2.9% vs. 3.3%; *p* = 0.025), this difference was not sustained following multivariable adjustment (aOR: 0.990 [0.900–1.090]; *p* = 0.844). VTE was more prevalent among patients with CS (6.1% vs. 4.8%; *p* < 0.001), an effect that persisted after adjustment (aOR: 1.252 [1.171–1.338]; *p* < 0.001). This increase was primarily driven by DVT (2.7% vs. 1.6%; *p* < 0.001; aOR: 1.644 [1.489–1.814]; *p* < 0.001), whereas PE (4.0% vs. 3.7%; *p* = 0.035) did not differ significantly in adjusted odds (aOR: 1.056 [0.974–1.146]; *p* = 0.186). Ischemic CVA rates demonstrated no significant difference (0.5% vs. 0.5%; *p* = 0.340), however, the adjusted model suggested significantly lower odds in the CS group (aOR: 0.748 [0.571–0.981]; *p* = 0.036). Conversely, TIAs were uncommon but slightly higher among CS admissions (0.3% vs. 0.1%; *p* < 0.001), with elevated odds persisting in multivariable analysis (aOR: 1.958 [1.587–2.402]; *p* < 0.001).

Discharge disposition patterns were broadly similar, with 59.6% of CS and 60.1% of non-CS admissions discharged routinely (*p* < 0.001). Moreover, resource utilization was notably higher in the CS group, evidenced by a prolonged median LOS (7 [IQR 4–12] vs. 5 [3–9] days; *p* = 0.009) and substantially greater total hospital charges (median $69,713 vs. $44,596; *p* < 0.001).

## Discussion

Building on the growing body of evidence around COVID-19 associated hyperinflammation, our study sought to clarify how varying degrees of CS severity influence patient outcomes in a large, nationally representative cohort. By systematically examining in-hospital mortality, acute cardiovascular events, cerebrovascular complications, cardiac arrhythmias, venous thromboembolism, and resource utilization, we observed gradients in both clinical impact and healthcare burden as CS severity advanced.

### In-hospital mortality

Mortality increased from 8.3% among non-CS patients to 26.9% in those with grade 4 CS, with both graded and dichotomized analyses demonstrating significantly higher adjusted odds of in-hospital death in CS cohorts compared to non-CS. These findings are consistent with Hu et al. (12) and Henderson et al. (2) who highlighted that hyperinflammatory states and unchecked cytokine release in COVID-19 lead to severe end-organ compromise, respiratory failure, multiorgan dysfunction, and increased mortality risk across diverse clinical settings. Our data further suggest that advancing CS grades may represent a critical clinical “tipping point” at which the cumulative burden of hyperinflammation outpaces compensatory mechanisms, a pattern attributed in prior mechanistic work to dysregulated cytokine networks, immune exhaustion, and progressive endothelial injury. Although our administrative dataset cannot evaluate pharmacologic therapy, randomized trials of the IL-6 receptor antagonist tocilizumab (RECOVERY, REMAP-CAP) (13) and the JAK1/2 inhibitor baricitinib (COV-BARRIER, ACTT-2) (14) have shown the greatest mortality benefit when these agents are initiated before life-threatening disease has developed, supporting the rationale for early identification of severe CS at the population level (15, 16). Sinha et al. proposed that early administration of immunomodulators could help avert disease progression in mild to moderate CS, potentially curbing mortality (6). Nonetheless, our results highlight the need for monitoring and treatment in advanced CS, given the association to increased mortality observed in these groups.

### Acute cardiovascular events

In the highest severity grade of CS, we observed an increase in the prevalence and odds of cardiac arrest, whereas cardiogenic shock remained infrequent despite increased odds in dichotomized analyses. Our results suggest that severe hyperinflammation may precipitate abrupt arrhythmogenic or hemodynamic collapse leading to cardiac arrest, while overt cardiogenic shock, typically tied to extensive myocardial dysfunction, may not be as commonly diagnosed or may be overshadowed by more fulminant presentations (17, 18). It is indeed plausible that patients experiencing a profound proinflammatory milieu in the most severe cases of CS may undergo sudden deterioration, culminating in catastrophic circulatory collapse before clinical shock states are clearly delineated, particularly when rapid decompensation results in early mortality or cardiac arrest.

In contrast, MI rates remained relatively stable, showing no significant adjusted difference between CS and non-CS groups. While SARS-CoV-2-associated endothelial dysfunction and prothrombotic pathways have been linked to higher MI risk (19, 20), It is possible that prophylactic anticoagulation strategies or acute care interventions, widely implemented during the pandemic, moderated the incidence of MI across groups (21). Nonetheless, the possibility of atypical or subclinical coronary events under severe inflammatory conditions warrants ongoing clinical surveillance.

HF incidence was lower in mild CS compared to non-CS but converged or slightly decreased at higher CS grades. In dichotomized analyses, CS was associated with reduced adjusted odds of HF overall. Several factors may explain this paradoxical pattern. Patients with chronic HF may have been less likely to be coded with CS due to overlapping clinical presentations; conversely, hyperinflammatory states may overshadow or mask HF exacerbations, leading to underdiagnosis or coding artifacts (22). Competing risks may further contribute, particularly in Grade 3 and Grade 4 cohorts, where in-hospital mortality reached 12.1% and 26.9%, respectively, as rapid progression to multiorgan failure or death may precede formal HF documentation. Treatment effects, including widespread corticosteroid and supportive care use, may additionally blunt the manifestations that would ordinarily prompt an HF code, while ICD-10 coding hierarchy may displace HF from secondary diagnoses when CS dominates the encounter. The apparent decline in HF is therefore more plausibly explained by the interplay of competing risks, treatment effects, and coding artifact than by a true protective effect. Prospective studies with biomarker and imaging data, particularly troponin, natriuretic peptides, and echocardiographic indices, will be essential to define HF risk more accurately in this setting.

### Cerebrovascular complications

Our analysis indicates that cerebrovascular events (Ischemic CVA and TIA) were relatively rare but displayed intriguing patterns. Dichotomized data showed a slight increase in TIA among CS patients, with persistently elevated adjusted odds, yet no significant rise in ischemic CVA. Graded analyses were limited by small event counts, especially in higher CS strata. These results partially resonate with previous observations by Merkler et al., who reported that stroke risk in COVID-19 is multifactorial and not solely attributable to hyperinflammation (23). Oxley et al. suggest that microvascular thrombosis and hypercoagulability can predispose to cerebrovascular events, although these processes may manifest more subtly (24).

Several pathophysiological mechanisms remain likely operative in this setting even when overt cerebrovascular events are administratively rare. Severe COVID-19–associated CS is characterized by direct viral and cytokine-mediated injury to the cerebral microvasculature, with platelet-fibrin microthrombus deposition and disruption of the blood-brain barrier. A distinct phenotype of COVID-19-associated stroke, with a higher proportion of large-vessel occlusions, multivessel territory infarcts, and cryptogenic mechanisms, has been linked to a hypercoagulable state involving elevations in D-dimer, fibrinogen, and plasminogen activator inhibitor-1 (25, 26). Importantly, much of this cerebrovascular burden is likely subclinical, manifesting as cerebral small-vessel disease or transient encephalopathy, and is systematically underrepresented when ascertainment depends on ICD-10 capture of overt stroke or TIA. The low overt event counts in our highest CS strata should therefore be interpreted with caution, and prospective studies with advanced neuroimaging and circulating biomarkers will be needed to delineate the true cerebrovascular footprint of severe CS.

### Cardiac arrhythmias

Cardiac arrhythmias, which we defined as a composite of atrial fibrillation and ventricular tachycardia/fibrillation, are recognized complications of severe COVID-19 (27). Notably, both graded and dichotomized analyses demonstrated declining incidence and odds of arrhythmias as CS severity increased. Although these findings may at first appear contradictory to prior studies, such as by Mohammad et al. (28), who documented arrhythmia surges in severely ill COVID-19 patients, they could reflect competing risks (e.g., rapid decompensation or death before arrhythmias are documented), differential coding practices in the setting of advanced CS, or treatment effects (e.g., use of corticosteroids, beta-blockers, or antiarrhythmics that may blunt arrhythmic triggers) (29). Continuous rhythm-monitoring data in critically ill or sedated patients may not be consistently translated into discharge codes, and ICD-10 coding hierarchy may further displace secondary arrhythmic diagnoses when CS dominates the encounter. Further prospective research with telemetry and continuous-monitoring data is therefore needed to clarify the true burden of arrhythmias within the CS spectrum.

### Venous thromboembolism

Our findings indicate an increase in VTE, encompassing PE and DVT, among patients with CS, with both dichotomized and graded analyses pointing to particularly high VTE risk in advanced CS. These results align with prior evidence that severe hyperinflammation disrupts multiple hemostatic mechanisms, producing a “prothrombotic storm.” For instance, Knight et al. observed that systemic inflammatory responses intensify coagulation cascades while reducing fibrinolysis, thereby increasing VTE rates (30). Similarly, our laboratory's recent work emphasizes that SARS-CoV-2–induced endothelial damage, platelet hyperactivation, and excessive cytokine release work in concert to promote clot formation, a process further exacerbated by impaired fibrinolysis due to upregulated plasminogen activator inhibitor-1 (31). Collectively, these data emphasize the necessity of proactive prophylactic anticoagulation and early risk stratification, particularly among individuals exhibiting advanced or rapidly escalating hyperinflammation, to limit VTE-related complications (32, 33).

### Resource utilization

Our findings indicate that resource utilization, as reflected by LOS and total hospital charges, rose disproportionately among patients with advanced CS. In graded analyses, LOS doubled relative to non-CS hospitalizations, and total charges increased considerably in grades 3 and 4. Likewise, in dichotomized analyses, all CS patients similarly exhibited extended LOS and higher overall costs. These findings are in line with previous reports indicating that COVID-19 lengthens critical care stays and that heightened inflammatory responses can demand substantial intensive care unit resources, underscoring the significant burden associated with advanced cases (34, 35).

### Immune-cardiac pathways and translational implications

Although our administrative dataset cannot resolve mechanism, the cardiovascular signal observed at the highest CS grades is biologically congruent with established cardiac immunopathology in severe COVID-19. IL-6 signaling through the JAK/STAT3 axis drives cardiomyocyte dysfunction and propagates vascular inflammation; TNF-α exerts direct negative inotropic effects and promotes endothelial activation; and IL-1β activates the NLRP3 inflammasome in cardiomyocytes and pericytes. Convergent activation of these pathways yields a coordinated prothrombotic and proinflammatory milieu that plausibly underlies the steep increases in venous thromboembolism and cardiac arrest seen at Grade 3 and Grade 4 in our cohort. Recent work in obese populations further implicates angiotensin II–driven cytokine amplification at the metabolic–immune interface (36), an axis particularly relevant given the high prevalence of obesity (37.8%–51.2%) we observed across CS cohorts. Each of these pathways is now therapeutically tractable, through IL-6 receptor antagonists, JAK inhibitors, and IL-1β blockade, providing a mechanistic anchor for the population-level associations reported here.

### Strengths and limitations

The strengths of our study are grounded in its use of the NIS, one of the largest, most comprehensive, and nationally representative inpatient databases in the United States. By evaluating a broad population of hospitalized adults with COVID-19 from diverse demographic and geographical backgrounds, our findings more accurately reflect real-world clinical practices and patient heterogeneity. This large-scale, population-level approach minimizes selection biases that may arise in single-center or narrower cohort studies, thereby significantly enhancing the generalizability of our results. Additionally, our graded classification of CS severity via standardized ICD-10 codes offers a more nuanced perspective than binary definitions alone. Finally, the application of multivariable logistic regression, adjusting for a wide spectrum of demographic, clinical, and hospital-level factors, bolsters the validity of observed associations between CS severity and in-hospital outcomes.

However, certain limitations merit consideration. As an administrative database, the NIS relies on ICD-10 coding for both exposures and outcomes, which may be subject to coding inaccuracies or misclassification. The diagnosis of CS itself remains variably defined in clinical practice, and the D89.83 sub-classifications, although clinically anchored to the Lee/ASTCT severity framework, do not directly capture serum cytokine concentrations (e.g., IL-6, TNF-α, IL-1β, IFN-γ), inflammatory biomarkers (CRP, ferritin, D-dimer), or measures of immune cell exhaustion; we are therefore unable to characterize cytokine profiles by severity grade or directly correlate specific cytokines with cardiovascular complications. Second, the NIS does not contain granular clinical information, such as laboratory markers, imaging findings, severity-of-illness scores, or specific pharmacologic therapies (e.g., corticosteroids, tocilizumab, sarilumab, baricitinib, anakinra, anticoagulants, antiarrhythmics), that could refine our understanding of the pathophysiological processes underlying CS or directly evaluate the impact of immunomodulatory interventions on the observed outcomes. Third, as an observational, cross-sectional analysis of a single year, our study cannot fully discern temporal relationships or causality (e.g., whether certain interventions preceded or followed disease progression). Fourth, despite adjustment for demographics, comorbidities, and hospital factors, the NIS lacks key confounders such as illness severity scores and medication use; their absence may partly explain the paradoxical associations concerning heart failure and arrhythmias in the CS cohort, where misclassification, competing risks, and treatment effects likely operate concurrently. Lastly, changes in COVID-19 variants and vaccination status during 2021 could impact the generalizability of these results to later periods; our data may not capture evolving management strategies or viral epidemiology in the ongoing pandemic.

## Conclusion

In this large, population-based analysis of hospitalized adults with COVID-19, we observed that advanced grades of CS, captured by ICD-10 D89.83 sub-classifications anchored to the Lee/ASTCT severity framework, were associated with increased mortality, cardiac arrest, VTE, and overall healthcare utilization. The graded signal across mortality and thromboembolic outcomes supports the prognostic value of this stratification scheme for population-level identification of high-risk patients and underscores the utility of routinely capturing CS severity in administrative records to enable scalable surveillance and risk stratification. Our findings suggest that severe hyperinflammation correlates with greater risk of adverse outcomes, including prolonged length of stay and higher hospitalization-related costs. Notably, certain cardiovascular outcomes (e.g., heart failure, arrhythmias) exhibited nuanced or even paradoxical trends in the presence of CS. Future prospective research incorporating detailed clinical, biomarker, and imaging data will be essential to refine therapeutic approaches and mitigate the healthcare burden of severe COVID-19–associated cytokine storm.

## Data Availability

The datasets presented in this article are not readily available because the data underlying this study were obtained from the 2021 National Inpatient Sample (NIS), a component of the Healthcare Cost and Utilization Project (HCUP) sponsored by the Agency for Healthcare Research and Quality. The NIS is publicly available for purchase through HCUP (https://hcup-us.ahrq.gov/) under a signed data use agreement. The authors are not permitted to distribute or share the NIS data directly, per the terms of the HCUP Data Use Agreement. Interested researchers may obtain the data independently through HCUP. Requests to access the datasets should be directed to Healthcare Cost and Utilization Project (HCUP), Agency for Healthcare Research and Quality, https://hcup-us.ahrq.gov/databases.jsp.
