# Supplementary material for: In-hospital cardiovascular outcomes of COVID-19-associated cytokine storm: a population-level study
Source: Front Epidemiol. 2026 Jun 3;6:1836720. doi: 10.3389/fepid.2026.1836720 (PMC13272423; doi:10.3389/fepid.2026.1836720)
Supplement: Supplementary file 1 [file Table1.docx]

**Supplementary Materials**

**S1 Table**

Sampling was based on the International Classification of Diseases, 10th Revision, Clinical Modification/Procedure Coding System (ICD-10 CM/PCS) codes

| **Initial Sample** | |
| --- | --- |
| **COVID-19** | ‘U09.9’ |

| **Cytokine Storm (“Cytokine Release Syndrome”)** | |
| --- | --- |
| **Cytokine Storm** | **- D89.831**: CRS, grade 1  **- D89.832**: CRS, grade 2  **- D89.833**: CRS, grade 3  **- D89.834**: CRS, grade 4  **- D89.839**: CRS, grade unspecified |

| **Exclusion Criteria** | |
| --- | --- |
| **1. Patients Who Have Received Chimeric Antigen Receptor T-cell (CAR-T) Therapy(w/CM codes)** | **Procedure Codes:** **-XW033C3:** Introduction of Engineered Autologous Chimeric Antigen Receptor T-cell  Immunotherapy into Peripheral Vein, Percutaneous Approach, New Technology Group 3  **-XW043C3:** Introduction of Engineered Autologous Chimeric Antigen Receptor T-cell  Immunotherapy into Central Vein, Percutaneous Approach, New Technology Group 3  **-XW23346:** Transfusion of Brexucabtagene Autoleucel Immunotherapy into Peripheral  Vein, Percutaneous Approach, New Technology Group 6  **-XW24346:** Transfusion of Brexucabtagene Autoleucel Immunotherapy into Central Vein,  Percutaneous Approach, New Technology Group 6  **-XW23376** – Transfusion of lisocabtagene maraleucel immunotherapy into peripheral  vein, percutaneous approach, new technology group 6  **-XW24376** – Transfusion of lisocabtagene maraleucel immunotherapy into central vein,  percutaneous approach, new technology 6  **Diagnosis codes**  **-Mantle Cell Lymphoma** (C83.11 - C83.19)  **-Diffuse Large B-Cell Lymphoma** (C83.31 - C83.39)  **-Unspecified B-Cell Lymphoma** (C85.11 - C85.19)  **-Mediastinal (Thymic) Large B-Cell Lymphoma** (C85.21 - C85.29)  **-Follicular Lymphoma Grade IIIb** (C82.41 - C82.49)  **-Acute Lymphoblastic Leukemia** (C91.00, C91.02)  **-Multiple Myeloma** (C90.00, C90.02) |
| **2. Patients Diagnosed with Hemophagocytic Lymphohistiocytosis (HLH) or Macrophage Activation Syndrome (MAS)** | - **D76.1**: Hemophagocytic lymphohistiocytosis - **D76.2**: Hemophagocytic syndrome, infection-associated - **M06.1**: Adult-onset Still's disease |
| **3. Patients with Graft-versus-Host Disease (GVHD) or Recent Stem Cell/Bone Marrow Transplant** | - **T86.00**: Unspecified complication of bone marrow transplant - **T86.01**: Bone marrow transplant rejection - **T86.02**: Bone marrow transplant failure - **T86.03**: Bone marrow transplant infection - **T86.09**: Other complications of bone marrow transplant - **Z94.81**: Bone marrow transplant status - **Z94.84**: Stem cells transplant status  **D89.810** Acute graft-versus-host disease  -**D89.811:** Chronic graft-versus-host disease  **-D89.812:** Acute on chronic graft-versus-host disease  **-D89.813:** unspecified |
| **4. Other Immunotherapies Known to Cause CRS** | -**Z51.12**: Encounter for antineoplastic immunotherapy **-Z92.22** Personal history of monoclonal drug therapy  **- Z92.26** Personal history of immune checkpoint inhibitor therapy  **-Z92.85** Personal history of cellular therapy  **-Z92.850** Personal history of Chimeric Antigen Receptor T-cell therapy  **-Z92.858** Personal history of other cellular therapy  -**Z92.86** Personal history of gene therapy |
| **5. Patients with non-COVID-19 Sepsis, Toxic Shock Syndrome, or Viral Infections Associated with Cytokine Storm** | - **A41.0**: Sepsis due to Staphylococcus aureus - **A41.1**: Sepsis due to other specified staphylococcus - **A41.2**: Sepsis due to unspecified staphylococcus - **A41.3**: Sepsis due to Hemophilus influenzae - **A41.4**: Sepsis due to anaerobes - **A41.50**: Gram-negative sepsis, unspecified - **A41.51**: Sepsis due to Escherichia coli (E. coli) - **A41.52**: Sepsis due to Pseudomonas - **A41.53**: Sepsis due to Serratia - **A41.59**: Other Gram-negative sepsis - **A41.81**: Sepsis due to Enterococcus - **A41.89**: Other specified sepsis - **A41.9**: Sepsis, unspecified organism - **A48.3**: Toxic shock syndrome - **B33.4**: Viral cardiomyopathy - **B33.24**: Viral myocarditis |

| **Comorbidities** | |
| --- | --- |
| **AIDS** | 'B20', 'O98711', 'O98712', 'O98713', 'O98719', 'O9872', 'O9873', 'Z21'] |
| **Alcohol abuse** | 'F1010', 'F1011', 'F10120', 'F10121', 'F10129', 'F10130', 'F10131', 'F10132', 'F10139', 'F1014', 'F10150', 'F10151', 'F10159', 'F10180', 'F10181', 'F10182', 'F10188', 'F1019', 'F1020', 'F1021', 'F10220', 'F10221', 'F10229', 'F10230', 'F10231', 'F10232', 'F10239', 'F1024', 'F10250', 'F10251', 'F10259', 'F1026', 'F1027', 'F10280', 'F10281', 'F10282', 'F10288', 'F1029', 'F1094', 'F10950', 'F10951', 'F10959', 'F1096', 'F1097', 'F10980', 'G621', 'I426', 'K2920', 'K2921', 'K7010', 'K7011', 'O99310', 'O99311', 'O99312', 'O99313', 'O99314', 'O99315' |
| **Autoimmune Disease** | 'A1801', 'A1802', 'A3984', 'A5441', 'A5442', 'L4050', 'L4051', 'L4054', 'L4059', 'L900', 'L940', 'L941', 'L943', 'M0500', 'M05011', 'M05012', 'M05019', 'M05021', 'M05022', 'M05029', 'M05031', 'M05032', 'M05039', 'M05041', 'M05042', 'M05049', 'M05051', 'M05052', 'M05059', 'M05061', 'M05062', 'M05069', 'M05071', 'M05072', 'M05079', 'M0509', 'M0510', 'M05111', 'M05112', 'M05119', 'M05121', 'M05122', 'M05129', 'M05131', 'M05132', 'M05139', 'M05141', 'M05142', 'M05149', 'M05151', 'M05152', 'M05159', 'M05161', 'M05162', 'M05169', 'M05171', 'M05172', 'M05179', 'M0519', 'M0520', 'M05211', 'M05212', 'M05219', 'M05221', 'M05222', 'M05229', 'M05231', 'M05232', 'M05239', 'M05241', 'M05242', 'M05249', 'M05251', 'M05252', 'M05259', 'M05261', 'M05262', 'M05269', 'M05271', 'M05272', 'M05279', 'M0529', 'M0530', 'M05311', 'M05312', 'M05319', 'M05321', 'M05322', 'M05329', 'M05331', 'M05332', 'M05339', 'M05341', 'M05342', 'M05349', 'M05351', 'M05352', 'M05359', 'M05361', 'M05362', 'M05369', 'M05371', 'M05372', 'M05379', 'M0539', 'M0540', 'M05411', 'M05412', 'M05419', 'M05421', 'M05422', 'M05429', 'M05431', 'M05432', 'M05439', 'M05441', 'M05442', 'M05449', 'M05451', 'M05452', 'M05459', 'M05461', 'M05462', 'M05469', 'M05471', 'M05472', 'M05479', 'M0549', 'M0550', 'M05511', 'M05512', 'M05519', 'M05521', 'M05522', 'M05529', 'M05531', 'M05532', 'M05539', 'M05541', 'M05542', 'M05549', 'M05551', 'M05552', 'M05559', 'M05561', 'M05562', 'M05569', 'M05571', 'M05572', 'M05579', 'M0559', 'M0560', 'M05611', 'M05612', 'M05619', 'M05621', 'M05622', 'M05629', 'M05631', 'M05632', 'M05639', 'M05641', 'M05642', 'M05649', 'M05651', 'M05652', 'M05659', 'M05661', 'M05662', 'M05669', 'M05671', 'M05672', 'M05679', 'M0569', 'M0570', 'M05711', 'M05712', 'M05719', 'M05721', 'M05722', 'M05729', 'M05731', 'M05732', 'M05739', 'M05741', 'M05742', 'M05749', 'M05751', 'M05752', 'M05759', 'M05761', 'M05762', 'M05769', 'M05771', 'M05772', 'M05779', 'M0579', 'M057A', 'M0580', 'M05811', 'M05812', 'M05819', 'M05821', 'M05822', 'M05829', 'M05831', 'M05832', 'M05839', 'M05841', 'M05842', 'M05849', 'M05851', 'M05852', 'M05859', 'M05861', 'M05862', 'M05869', 'M05871', 'M05872', 'M05879', 'M0589', 'M058A', 'M059', 'M0600', 'M06011', 'M06012', 'M06019', 'M06021', 'M06022', 'M06029', 'M06031', 'M06032', 'M06039', 'M06041', 'M06042', 'M06049', 'M06051', 'M06052', 'M06059', 'M06061', 'M06062', 'M06069', 'M06071', 'M06072', 'M06079', 'M0608', 'M0609', 'M060A', 'M061', 'M0620', 'M06211', 'M06212', 'M06219', 'M06221', 'M06222', 'M06229', 'M06231', 'M06232', 'M06239', 'M06241', 'M06242', 'M06249', 'M06251', 'M06252', 'M06259', 'M06261', 'M06262', 'M06269', 'M06271', 'M06272', 'M06279', 'M0628', 'M0629', 'M0630', 'M06311', 'M06312', 'M06319', 'M06321', 'M06322', 'M06329', 'M06331', 'M06332', 'M06339', 'M06341', 'M06342', 'M06349', 'M06351', 'M06352', 'M06359', 'M06361', 'M06362', 'M06369', 'M06371', 'M06372', 'M06379', 'M0638', 'M0639', 'M064', 'M0680', 'M06811', 'M06812', 'M06819', 'M06821', 'M06822', 'M06829', 'M06831', 'M06832', 'M06839', 'M06841', 'M06842', 'M06849', 'M06851', 'M06852', 'M06859', 'M06861', 'M06862', 'M06869', 'M06871', 'M06872', 'M06879', 'M0688', 'M0689', 'M068A', 'M069', 'M0760', 'M07611', 'M07612', 'M07619', 'M07621', 'M07622', 'M07629', 'M07631', 'M07632', 'M07639', 'M07641', 'M07642', 'M07649', 'M07651', 'M07652', 'M07659', 'M07661', 'M07662', 'M07669', 'M07671', 'M07672', 'M07679', 'M0768', 'M0769', 'M0800', 'M08011', 'M08012', 'M08019', 'M08021', 'M08022', 'M08029', 'M08031', 'M08032', 'M08039', 'M08041', 'M08042', 'M08049', 'M08051', 'M08052', 'M08059', 'M08061', 'M08062', 'M08069', 'M08071', 'M08072', 'M08079', 'M0808', 'M0809', 'M080A', 'M081', 'M0820', 'M08211', 'M08212', 'M08219', 'M08221', 'M08222', 'M08229', 'M08231', 'M08232', 'M08239', 'M08241', 'M08242', 'M08249', 'M08251', 'M08252', 'M08259', 'M08261', 'M08262', 'M08269', 'M08271', 'M08272', 'M08279', 'M0828', 'M0829', 'M082A', 'M083', 'M0840', 'M08411', 'M08412', 'M08419', 'M08421', 'M08422', 'M08429', 'M08431', 'M08432', 'M08439', 'M08441', 'M08442', 'M08449', 'M08451', 'M08452', 'M08459', 'M08461', 'M08462', 'M08469', 'M08471', 'M08472', 'M08479', 'M0848', 'M084A', 'M0880', 'M08811', 'M08812', 'M08819', 'M08821', 'M08822', 'M08829', 'M08831', 'M08832', 'M08839', 'M08841', 'M08842', 'M08849', 'M08851', 'M08852', 'M08859', 'M08861', 'M08862', 'M08869', 'M08871', 'M08872', 'M08879', 'M0888', 'M0889', 'M0890', 'M08911', 'M08912', 'M08919', 'M08921', 'M08922', 'M08929', 'M08931', 'M08932', 'M08939', 'M08941', 'M08942', 'M08949', 'M08951', 'M08952', 'M08959', 'M08961', 'M08962', 'M08969', 'M08971', 'M08972', 'M08979', 'M0898', 'M0899', 'M089A', 'M1200', 'M12011', 'M12012', 'M12019', 'M12021', 'M12022', 'M12029', 'M12031', 'M12032', 'M12039', 'M12041', 'M12042', 'M12049', 'M12051', 'M12052', 'M12059', 'M12061', 'M12062', 'M12069', 'M12071', 'M12072', 'M12079', 'M1208', 'M1209', 'M300', 'M301', 'M302', 'M303', 'M308', 'M310', 'M311', 'M3110', 'M3111', 'M3119', 'M312', 'M3130', 'M3131', 'M314', 'M315', 'M316', 'M317', 'M318', 'M319', 'M320', 'M3210', 'M3211', 'M3212', 'M3213', 'M3214', 'M3215', 'M3219', 'M328', 'M329', 'M3300', 'M3301', 'M3302', 'M3303', 'M3309', 'M3310', 'M3311', 'M3312', 'M3313', 'M3319', 'M3320', 'M3321', 'M3322', 'M3329', 'M3390', 'M3391', 'M3392', 'M3393', 'M3399', 'M340', 'M341', 'M342', 'M3481', 'M3482', 'M3483', 'M3489', 'M349', 'M3500', 'M3501', 'M3502', 'M3503', 'M3504', 'M3505', 'M3506', 'M3507', 'M3508', 'M3509', 'M350A', 'M350B', 'M350C', 'M351', 'M352', 'M353', 'M355', 'M356', 'M358', 'M3581', 'M3589', 'M359', 'M360', 'M361', 'M368', 'M450', 'M451', 'M452', 'M453', 'M454', 'M455', 'M456', 'M457', 'M458', 'M459', 'M45A0', 'M45A1', 'M45A2', 'M45A3', 'M45A4', 'M45A5', 'M45A6', 'M45A7', 'M45A8', 'M45AB' |
| **Anemia** | 'D500' |
| **Metastatic cancer** | 'C770', 'C771', 'C772', 'C773', 'C774', 'C775', 'C778', 'C779', 'C7800', 'C7801', 'C7802', 'C781', 'C782', 'C7830', 'C7839', 'C784', 'C785', 'C786', 'C787', 'C7880', 'C7889', 'C7900', 'C7901', 'C7902', 'C7910', 'C7911', 'C7919', 'C792', 'C7931', 'C7932', 'C7940', 'C7949', 'C7951', 'C7952', 'C7960', 'C7961', 'C7962', 'C7963', 'C7970', 'C7971', 'C7972', 'C7981', 'C7982', 'C7989', 'C799', 'C7B00', 'C7B01', 'C7B02', 'C7B03', 'C7B04', 'C7B09', 'C7B1', 'C7B8', 'C800' |
| **Solid Cancer** | ['C000', 'C001', 'C002', 'C003', 'C004', 'C005', 'C006', 'C008', 'C009', 'C01', 'C020', 'C021', 'C022', 'C023', 'C024', 'C028', 'C029', 'C030', 'C031', 'C039', 'C040', 'C041', 'C048', 'C049', 'C050', 'C051', 'C052', 'C058', 'C059', 'C060', 'C061', 'C062', 'C0680', 'C0689', 'C069', 'C07', 'C080', 'C081', 'C089', 'C090', 'C091', 'C098', 'C099', 'C100', 'C101', 'C102', 'C103', 'C104', 'C108', 'C109', 'C110', 'C111', 'C112', 'C113', 'C118', 'C119', 'C12', 'C130', 'C131', 'C132', 'C138', 'C139', 'C140', 'C142', 'C148', 'C153', 'C154', 'C155', 'C158', 'C159', 'C160', 'C161', 'C162', 'C163', 'C164', 'C165', 'C166', 'C168', 'C169', 'C170', 'C171', 'C172', 'C173', 'C178', 'C179', 'C180', 'C181', 'C182', 'C183', 'C184', 'C185', 'C186', 'C187', 'C188', 'C189', 'C19', 'C20', 'C210', 'C211', 'C212', 'C218', 'C220', 'C221', 'C222', 'C223', 'C224', 'C227', 'C228', 'C229', 'C23', 'C240', 'C241', 'C248', 'C249', 'C250', 'C251', 'C252', 'C253', 'C254', 'C257', 'C258', 'C259', 'C260', 'C261', 'C269', 'C300', 'C301', 'C310', 'C311', 'C312', 'C313', 'C318', 'C319', 'C320', 'C321', 'C322', 'C323', 'C328', 'C329', 'C33', 'C3400', 'C3401', 'C3402', 'C3410', 'C3411', 'C3412', 'C342', 'C3430', 'C3431', 'C3432', 'C3480', 'C3481', 'C3482', 'C3490', 'C3491', 'C3492', 'C37', 'C380', 'C381', 'C382', 'C383', 'C384', 'C388', 'C390', 'C399', 'C4000', 'C4001', 'C4002', 'C4010', 'C4011', 'C4012', 'C4020', 'C4021', 'C4022', 'C4030', 'C4031', 'C4032', 'C4080', 'C4081', 'C4082', 'C4090', 'C4091', 'C4092', 'C410', 'C411', 'C412', 'C413', 'C414', 'C419', 'C430', 'C4310', 'C4311', 'C43111', 'C43112', 'C4312', 'C43121', 'C43122', 'C4320', 'C4321', 'C4322', 'C4330', 'C4331', 'C4339', 'C434', 'C4351', 'C4352', 'C4359', 'C4360', 'C4361', 'C4362', 'C4370', 'C4371', 'C4372', 'C438', 'C439', 'C4400', 'C4409', 'C44101', 'C44102', 'C441021', 'C441022', 'C44109', 'C441091', 'C441092', 'C44131', 'C441321', 'C441322', 'C441391', 'C441392', 'C44191', 'C44192', 'C441921', 'C441922', 'C44199', 'C441991', 'C441992', 'C44201', 'C44202', 'C44209', 'C44291', 'C44292', 'C44299', 'C44300', 'C44301', 'C44309', 'C44390', 'C44391', 'C44399', 'C4440', 'C4449', 'C44500', 'C44501', 'C44509', 'C44590', 'C44591', 'C44599', 'C44601', 'C44602', 'C44609', 'C44691', 'C44692', 'C44699', 'C44701', 'C44702', 'C44709', 'C44791', 'C44792', 'C44799', 'C4480', 'C4489', 'C4490', 'C4499', 'C450', 'C451', 'C452', 'C457', 'C459', 'C460', 'C461', 'C462', 'C463', 'C464', 'C4650', 'C4651', 'C4652', 'C467', 'C469', 'C470', 'C4710', 'C4711', 'C4712', 'C4720', 'C4721', 'C4722', 'C473', 'C474', 'C475', 'C476', 'C478', 'C479', 'C480', 'C481', 'C482', 'C488', 'C490', 'C4910', 'C4911', 'C4912', 'C4920', 'C4921', 'C4922', 'C493', 'C494', 'C495', 'C496', 'C498', 'C499', 'C49A0', 'C49A1', 'C49A2', 'C49A3', 'C49A4', 'C49A5', 'C49A9', 'C4A0', 'C4A10', 'C4A11', 'C4A111', 'C4A112', 'C4A12', 'C4A121', 'C4A122', 'C4A20', 'C4A21', 'C4A22', 'C4A30', 'C4A31', 'C4A39', 'C4A4', 'C4A51', 'C4A52', 'C4A59', 'C4A60', 'C4A61', 'C4A62', 'C4A70', 'C4A71', 'C4A72', 'C4A8', 'C4A9', 'C50011', 'C50012', 'C50019', 'C50021', 'C50022', 'C50029', 'C50111', 'C50112', 'C50119', 'C50121', 'C50122', 'C50129', 'C50211', 'C50212', 'C50219', 'C50221', 'C50222', 'C50229', 'C50311', 'C50312', 'C50319', 'C50321', 'C50322', 'C50329', 'C50411', 'C50412', 'C50419', 'C50421', 'C50422', 'C50429', 'C50511', 'C50512', 'C50519', 'C50521', 'C50522', 'C50529', 'C50611', 'C50612', 'C50619', 'C50621', 'C50622', 'C50629', 'C50811', 'C50812', 'C50819', 'C50821', 'C50822', 'C50829', 'C50911', 'C50912', 'C50919', 'C50921', 'C50922', 'C50929', 'C510', 'C511', 'C512', 'C518', 'C519', 'C52', 'C530', 'C531', 'C538', 'C539', 'C540', 'C541', 'C542', 'C543', 'C548', 'C549', 'C55', 'C561', 'C562', 'C563', 'C569', 'C5700', 'C5701', 'C5702', 'C5710', 'C5711', 'C5712', 'C5720', 'C5721', 'C5722', 'C573', 'C574', 'C577', 'C578', 'C579', 'C58', 'C600', 'C601', 'C602', 'C608', 'C609', 'C61', 'C6200', 'C6201', 'C6202', 'C6210', 'C6211', 'C6212', 'C6290', 'C6291', 'C6292', 'C6300', 'C6301', 'C6302', 'C6310', 'C6311', 'C6312', 'C632', 'C637', 'C638', 'C639', 'C641', 'C642', 'C649', 'C651', 'C652', 'C659', 'C661', 'C662', 'C669', 'C670', 'C671', 'C672', 'C673', 'C674', 'C675', 'C676', 'C677', 'C678', 'C679', 'C680', 'C681', 'C688', 'C689', 'C6900', 'C6901', 'C6902', 'C6910', 'C6911', 'C6912', 'C6920', 'C6921', 'C6922', 'C6930', 'C6931', 'C6932', 'C6940', 'C6941', 'C6942', 'C6950', 'C6951', 'C6952', 'C6960', 'C6961', 'C6962', 'C6980', 'C6981', 'C6982', 'C6990', 'C6991', 'C6992', 'C700', 'C701', 'C709', 'C710', 'C711', 'C712', 'C713', 'C714', 'C715', 'C716', 'C717', 'C718', 'C719', 'C720', 'C721', 'C7220', 'C7221', 'C7222', 'C7230', 'C7231', 'C7232', 'C7240', 'C7241', 'C7242', 'C7250', 'C7259', 'C729', 'C73', 'C7400', 'C7401', 'C7402', 'C7410', 'C7411', 'C7412', 'C7490', 'C7491', 'C7492', 'C750', 'C751', 'C752', 'C753', 'C754', 'C755', 'C758', 'C759', 'C760', 'C761', 'C762', 'C763', 'C7640', 'C7641', 'C7642', 'C7650', 'C7651', 'C7652', 'C768', 'C7A00', 'C7A010', 'C7A011', 'C7A012', 'C7A019', 'C7A020', 'C7A021', 'C7A022', 'C7A023', 'C7A024', 'C7A025', 'C7A026', 'C7A029', 'C7A090', 'C7A091', 'C7A092', 'C7A093', 'C7A094', 'C7A095', 'C7A096', 'C7A098', 'C7A1', 'C7A8', 'D469', 'E3121', 'E3122', 'E3123' |
| **Cerebrovascular disease** | 'G450', 'G451', 'G452', 'G453', 'G454', 'G458', 'G459', 'G460', 'G461', 'G462', 'G463', 'G464', 'G465', 'G466', 'G467', 'G468', 'H3400', 'H3401', 'H3402', 'H3403', 'H3410', 'H3411', 'H3412', 'H3413', 'H34211', 'H34212', 'H34213', 'H34219', 'H34231', 'H34232', 'H34233', 'H34239', 'I6000', 'I6001', 'I6002', 'I6010', 'I6011', 'I6012', 'I602', 'I6020', 'I6021', 'I6022', 'I6030', 'I6031', 'I6032', 'I604', 'I6050', 'I6051', 'I6052', 'I606', 'I607', 'I608', 'I609', 'I610', 'I611', 'I612', 'I613', 'I614', 'I615', 'I616', 'I618', 'I619', 'I6200', 'I6203', 'I621', 'I629', 'I6300', 'I63011', 'I63012', 'I63013', 'I63019', 'I6302', 'I63031', 'I63032', 'I63033', 'I63039', 'I6309', 'I6310', 'I63111', 'I63112', 'I63113', 'I63119', 'I6312', 'I63131', 'I63132', 'I63133', 'I63139', 'I6319', 'I6320', 'I63211', 'I63212', 'I63213', 'I63219', 'I6322', 'I63231', 'I63232', 'I63233', 'I63239', 'I6329', 'I6330', 'I63311', 'I63312', 'I63313', 'I63319', 'I63321', 'I63322', 'I63323', 'I63329', 'I63331', 'I63332', 'I63333', 'I63339', 'I63341', 'I63342', 'I63343', 'I63349', 'I6339', 'I6340', 'I63411', 'I63412', 'I63413', 'I63419', 'I63421', 'I63422', 'I63423', 'I63429', 'I63431', 'I63432', 'I63433', 'I63439', 'I63441', 'I63442', 'I63443', 'I63449', 'I6349', 'I6350', 'I63511', 'I63512', 'I63513', 'I63519', 'I63521', 'I63522', 'I63523', 'I63529', 'I63531', 'I63532', 'I63533', 'I63539', 'I63541', 'I63542', 'I63543', 'I63549', 'I6359', 'I636', 'I638', 'I6381', 'I6389', 'I639', 'I6501', 'I6502', 'I6503', 'I6509', 'I651', 'I6521', 'I6522', 'I6523', 'I6529', 'I658', 'I659', 'I6601', 'I6602', 'I6603', 'I6609', 'I6611', 'I6612', 'I6613', 'I6619', 'I6621', 'I6622', 'I6623', 'I6629', 'I663', 'I668', 'I669' |
| **Sequelae of Cerebrovascular Disease** | 'I6930', 'I6931', 'I69310', 'I69311', 'I69312', 'I69313', 'I69314', 'I69315', 'I69318', 'I69319', 'I69320', 'I69321', 'I69322', 'I69323', 'I69328', 'I69331', 'I69332', 'I69333', 'I69334', 'I69339', 'I69341', 'I69342', 'I69343', 'I69344', 'I69349', 'I69351', 'I69352', 'I69353', 'I69354', 'I69359', 'I69361', 'I69362', 'I69363', 'I69364', 'I69365', 'I69369', 'I69390', 'I69391', 'I69392', 'I69393', 'I69398', 'I6980', 'I6981', 'I69810', 'I69811', 'I69812', 'I69813', 'I69814', 'I69815', 'I69818', 'I69819', 'I69820', 'I69821', 'I69822', 'I69823', 'I69828', 'I69831', 'I69832', 'I69833', 'I69834', 'I69839', 'I69841', 'I69842', 'I69843', 'I69844', 'I69849', 'I69851', 'I69852', 'I69853', 'I69854', 'I69859', 'I69861', 'I69862', 'I69863', 'I69864', 'I69865', 'I69869', 'I69890', 'I69891', 'I69892', 'I69893', 'I69898', 'I6990', 'I6991', 'I69910', 'I69911', 'I69912', 'I69913', 'I69914', 'I69915', 'I69918', 'I69919', 'I69920', 'I69921', 'I69922', 'I69923', 'I69928', 'I69931', 'I69932', 'I69933', 'I69934', 'I69939', 'I69941', 'I69942', 'I69943', 'I69944', 'I69949', 'I69951', 'I69952', 'I69953', 'I69954', 'I69959', 'I69961', 'I69962', 'I69963', 'I69964', 'I69965', 'I69969', 'I69990', 'I69991', 'I69992', 'I69993', 'I69998', 'P91821', 'P91822', 'P91823', 'P91829' |
| **Coagulopathy** | 'D6102', 'D6109', 'D611', 'D612', 'D613', 'D61810', 'D61811', 'D61818', 'D6182', 'D6189', 'D619', 'D65', 'D66', 'D67', 'D680', 'D6800', 'D6801', 'D68020', 'D68021', 'D68022', 'D68023', 'D68029', 'D6803', 'D6804', 'D6809', 'D681', 'D682', 'D68311', 'D68312', 'D68318', 'D6832', 'D684', 'D688', 'D689', 'D691', 'D693', 'D6941', 'D6942', 'D6949', 'D6951', 'D6959', 'D696', 'D698', 'D699', 'D7582', 'D75821', 'D75822', 'D75828', 'D75829', 'D7584', 'O99111', 'O99112', 'O99113', 'O99119', 'O9912', 'O9913' |
| **Dementia** | 'F0150', 'F0151', 'F01511', 'F01518', 'F0152', 'F0153', 'F0154', 'F01A0', 'F01A11', 'F01A18', 'F01A2', 'F01A3', 'F01A4', 'F01B0', 'F01B11', 'F01B18', 'F01B2', 'F01B3', 'F01B4', 'F01C0', 'F01C11', 'F01C18', 'F01C2', 'F01C3', 'F01C4', 'F0280', 'F0281', 'F02811', 'F02818', 'F0282', 'F0283', 'F0284', 'F02A0', 'F02A11', 'F02A18', 'F02A2', 'F02A3', 'F02A4', 'F02B0', 'F02B11', 'F02B18', 'F02B2', 'F02B3', 'F02B4', 'F02C0', 'F02C11', 'F02C18', 'F02C2', 'F02C3', 'F02C4', 'F0390', 'F0391', 'F03911', 'F03918', 'F0392', 'F0393', 'F0394', 'F03A0', 'F03A11', 'F03A18', 'F03A2', 'F03A3', 'F03A4', 'F03B0', 'F03B11', 'F03B18', 'F03B2', 'F03B3', 'F03B4', 'F03C0', 'F03C11', 'F03C18', 'F03C2', 'F03C3', 'F03C4', 'F0670', 'F0671', 'G300', 'G301', 'G308', 'G309', 'G3101', 'G3109', 'G311', 'G312', 'G3180', 'G3181', 'G3182', 'G3183', 'G3185', 'G3186', 'G3189', 'G319' |
| **Depression** | 'F0631', 'F0632', 'F0634', 'F320', 'F321', 'F322', 'F323', 'F328', 'F3281', 'F3289', 'F329', 'F32A', 'F330', 'F331', 'F332', 'F333', 'F338', 'F339', 'F341' |
| **Diabetes, Complicated** | ['E0821', 'E0822', 'E0829', 'E08311', 'E08319', 'E08321', 'E083211', 'E083212', 'E083213', 'E083219', 'E08329', 'E083291', 'E083292', 'E083293', 'E083299', 'E08331', 'E083311', 'E083312', 'E083313', 'E083319', 'E08339', 'E083391', 'E083392', 'E083393', 'E083399', 'E08341', 'E083411', 'E083412', 'E083413', 'E083419', 'E08349', 'E083491', 'E083492', 'E083493', 'E083499', 'E08351', 'E083511', 'E083512', 'E083513', 'E083519', 'E083521', 'E083522', 'E083523', 'E083529', 'E083531', 'E083532', 'E083533', 'E083539', 'E083541', 'E083542', 'E083543', 'E083549', 'E083551', 'E083552', 'E083553', 'E083559', 'E08359', 'E083591', 'E083592', 'E083593', 'E083599', 'E0836', 'E0837X1', 'E0837X2', 'E0837X3', 'E0837X9', 'E0839', 'E0840', 'E0841', 'E0842', 'E0843', 'E0844', 'E0849', 'E0851', 'E0852', 'E0859', 'E08610', 'E08618', 'E08620', 'E08621', 'E08622', 'E08628', 'E08630', 'E08638', 'E08641', 'E08649', 'E0865', 'E0869', 'E088', 'E0921', 'E0922', 'E0929', 'E09311', 'E09319', 'E09321', 'E093211', 'E093212', 'E093213', 'E093219', 'E09329', 'E093291', 'E093292', 'E093293', 'E093299', 'E09331', 'E093311', 'E093312', 'E093313', 'E093319', 'E09339', 'E093391', 'E093392', 'E093393', 'E093399', 'E09341', 'E093411', 'E093412', 'E093413', 'E093419', 'E09349', 'E093491', 'E093492', 'E093493', 'E093499', 'E09351', 'E093511', 'E093512', 'E093513', 'E093519', 'E093521', 'E093522', 'E093523', 'E093529', 'E093531', 'E093532', 'E093533', 'E093539', 'E093541', 'E093542', 'E093543', 'E093549', 'E093551', 'E093552', 'E093553', 'E093559', 'E09359', 'E093591', 'E093592', 'E093593', 'E093599', 'E0936', 'E0937X1', 'E0937X2', 'E0937X3', 'E0937X9', 'E0939', 'E0940', 'E0941', 'E0942', 'E0943', 'E0944', 'E0949', 'E0951', 'E0952', 'E0959', 'E09610', 'E09618', 'E09620', 'E09621', 'E09622', 'E09628', 'E09630', 'E09638', 'E09641', 'E09649', 'E0965', 'E0969', 'E098', 'E1021', 'E1022', 'E1029', 'E10311', 'E10319', 'E10321', 'E103211', 'E103212', 'E103213', 'E103219', 'E10329', 'E103291', 'E103292', 'E103293', 'E103299', 'E10331', 'E103311', 'E103312', 'E103313', 'E103319', 'E10339', 'E103391', 'E103392', 'E103393', 'E103399', 'E10341', 'E103411', 'E103412', 'E103413', 'E103419', 'E10349', 'E103491', 'E103492', 'E103493', 'E103499', 'E10351', 'E103511', 'E103512', 'E103513', 'E103519', 'E103521', 'E103522', 'E103523', 'E103529', 'E103531', 'E103532', 'E103533', 'E103539', 'E103541', 'E103542', 'E103543', 'E103549', 'E103551', 'E103552', 'E103553', 'E103559', 'E10359', 'E103591', 'E103592', 'E103593', 'E103599', 'E1036', 'E1037X1', 'E1037X2', 'E1037X3', 'E1037X9', 'E1039', 'E1040', 'E1041', 'E1042', 'E1043', 'E1044', 'E1049', 'E1051', 'E1052', 'E1059', 'E10610', 'E10618', 'E10620', 'E10621', 'E10622', 'E10628', 'E10630', 'E10638', 'E10641', 'E10649', 'E1065', 'E1069', 'E108', 'E1121', 'E1122', 'E1129', 'E11311', 'E11319', 'E11321', 'E113211', 'E113212', 'E113213', 'E113219', 'E11329', 'E113291', 'E113292', 'E113293', 'E113299', 'E11331', 'E113311', 'E113312', 'E113313', 'E113319', 'E11339', 'E113391', 'E113392', 'E113393', 'E113399', 'E11341', 'E113411', 'E113412', 'E113413', 'E113419', 'E11349', 'E113491', 'E113492', 'E113493', 'E113499', 'E11351', 'E113511', 'E113512', 'E113513', 'E113519', 'E113521', 'E113522', 'E113523', 'E113529', 'E113531', 'E113532', 'E113533', 'E113539', 'E113541', 'E113542', 'E113543', 'E113549', 'E113551', 'E113552', 'E113553', 'E113559', 'E11359', 'E113591', 'E113592', 'E113593', 'E113599', 'E1136', 'E1137X1', 'E1137X2', 'E1137X3', 'E1137X9', 'E1139', 'E1140', 'E1141', 'E1142', 'E1143', 'E1144', 'E1149', 'E1151', 'E1152', 'E1159', 'E11610', 'E11618', 'E11620', 'E11621', 'E11622', 'E11628', 'E11630', 'E11638', 'E11641', 'E11649', 'E1165', 'E1169', 'E118', 'E1321', 'E1322', 'E1329', 'E13311', 'E13319', 'E13321', 'E133211', 'E133212', 'E133213', 'E133219', 'E13329', 'E133291', 'E133292', 'E133293', 'E133299', 'E13331', 'E133311', 'E133312', 'E133313', 'E133319', 'E13339', 'E133391', 'E133392', 'E133393', 'E133399', 'E13341', 'E133411', 'E133412', 'E133413', 'E133419', 'E13349', 'E133491', 'E133492', 'E133493', 'E133499', 'E13351', 'E133511', 'E133512', 'E133513', 'E133519', 'E133521', 'E133522', 'E133523', 'E133529', 'E133531', 'E133532', 'E133533', 'E133539', 'E133541', 'E133542', 'E133543', 'E133549', 'E133551', 'E133552', 'E133553', 'E133559', 'E13359', 'E133591', 'E133592', 'E133593', 'E133599', 'E1336', 'E1337X1', 'E1337X2', 'E1337X3', 'E1337X9', 'E1339', 'E1340', 'E1341', 'E1342', 'E1343', 'E1344', 'E1349', 'E1351', 'E1352', 'E1359', 'E13610', 'E13618', 'E13620', 'E13621', 'E13622', 'E13628', 'E13630', 'E13638', 'E13641', 'E13649', 'E1365', 'E1369', 'E138' |
| **Diabetes, uncomplicated** | 'E0800', 'E0801', 'E0810', 'E0811', 'E089', 'E0900', 'E0901', 'E0910', 'E0911', 'E099', 'E1010', 'E1011', 'E109', 'E1100', 'E1101', 'E1110', 'E1111', 'E119', 'E1300', 'E1301', 'E1310', 'E1311', 'E139', 'O24011', 'O24012', 'O24013', 'O24019', 'O2402', 'O2403', 'O24111', 'O24112', 'O24113', 'O24119', 'O2412', 'O2413', 'O24311', 'O24312', 'O24313', 'O24319', 'O2432', 'O2433', 'O24410', 'O24414', 'O24415', 'O24419', 'O24420', 'O24424', 'O24425', 'O24429', 'O24430', 'O24434', 'O24435', 'O24439', 'O24811', 'O24812', 'O24813', 'O24819', 'O2482', 'O2483', 'O24911', 'O24912', 'O24913', 'O24919', 'O2492', 'O2493' |
| **Drug abuse** | 'F1110', 'F1111', 'F11120', 'F11121', 'F11122', 'F11129', 'F1113', 'F1114', 'F11150', 'F11151', 'F11159', 'F11181', 'F11182', 'F11188', 'F1119', 'F1120', 'F1121', 'F11220', 'F11221', 'F11222', 'F11229', 'F1123', 'F1124', 'F11250', 'F11251', 'F11259', 'F11281', 'F11282', 'F11288', 'F1129', 'F1210', 'F1211', 'F12120', 'F12121', 'F12122', 'F12129', 'F1213', 'F12150', 'F12151', 'F12159', 'F12180', 'F12188', 'F1219', 'F1220', 'F1221', 'F12220', 'F12221', 'F12222', 'F12229', 'F1223', 'F12250', 'F12251', 'F12259', 'F12280', 'F12288', 'F1229', 'F1310', 'F1311', 'F13120', 'F13121', 'F13129', 'F13130', 'F13131', 'F13132', 'F13139', 'F1314', 'F13150', 'F13151', 'F13159', 'F13180', 'F13181', 'F13182', 'F13188', 'F1319', 'F1320', 'F1321', 'F13220', 'F13221', 'F13229', 'F13230', 'F13231', 'F13232', 'F13239', 'F1324', 'F13250', 'F13251', 'F13259', 'F1326', 'F1327', 'F13280', 'F13281', 'F13282', 'F13288', 'F1329', 'F1410', 'F1411', 'F14120', 'F14121', 'F14122', 'F14129', 'F1413', 'F1414', 'F14150', 'F14151', 'F14159', 'F14180', 'F14181', 'F14182', 'F14188', 'F1419', 'F1420', 'F1421', 'F14220', 'F14221', 'F14222', 'F14229', 'F1423', 'F1424', 'F14250', 'F14251', 'F14259', 'F14280', 'F14281', 'F14282', 'F14288', 'F1429', 'F1510', 'F1511', 'F15120', 'F15121', 'F15122', 'F15129', 'F1513', 'F1514', 'F15150', 'F15151', 'F15159', 'F15180', 'F15181', 'F15182', 'F15188', 'F1519', 'F1520', 'F1521', 'F15220', 'F15221', 'F15222', 'F15229', 'F1523', 'F1524', 'F15250', 'F15251', 'F15259', 'F15280', 'F15281', 'F15282', 'F15288', 'F1529', 'F1610', 'F1611', 'F16120', 'F16121', 'F16122', 'F16129', 'F1614', 'F16150', 'F16151', 'F16159', 'F16180', 'F16183', 'F16188', 'F1619', 'F1620', 'F1621', 'F16220', 'F16221', 'F16229', 'F1624', 'F16250', 'F16251', 'F16259', 'F16280', 'F16283', 'F16288', 'F1629', 'F1810', 'F1811', 'F18120', 'F18121', 'F18129', 'F1814', 'F18150', 'F18151', 'F18159', 'F1817', 'F18180', 'F18188', 'F1819', 'F1820', 'F1821', 'F18220', 'F18221', 'F18229', 'F1824', 'F18250', 'F18251', 'F18259', 'F1827', 'F18280', 'F18288', 'F1829', 'F1910', 'F1911', 'F19120', 'F19121', 'F19122', 'F19129', 'F19130', 'F19131', 'F19132', 'F19139', 'F1914', 'F19150', 'F19151', 'F19159', 'F1916', 'F1917', 'F19180', 'F19181', 'F19182', 'F19188', 'F1919', 'F1920', 'F1921', 'F19220', 'F19221', 'F19222', 'F19229', 'F19230', 'F19231', 'F19232', 'F19239', 'F1924', 'F19250', 'F19251', 'F19259', 'F1926', 'F1927', 'F19280', 'F19281', 'F19282', 'F19288', 'F1929', 'O99320', 'O99321', 'O99322', 'O99323', 'O99324', 'O99325' |
| **Hypertension,**  **complicated** | 'H35031', 'H35032', 'H35033', 'H35039', 'I110', 'I119', 'I120', 'I129', 'I130', 'I1310', 'I1311', 'I132', 'I150', 'I151', 'I152', 'I158', 'I159', 'I161', 'I674', 'O10111', 'O10112', 'O10113', 'O10119', 'O1012', 'O1013', 'O10211', 'O10212', 'O10213', 'O10219', 'O1022', 'O1023', 'O10311', 'O10312', 'O10313', 'O10319', 'O1032', 'O1033', 'O10411', 'O10412', 'O10413', 'O10419', 'O1042', 'O1043', 'O10911', 'O10912', 'O10913', 'O10919', 'O1092', 'O1093', 'O111', 'O112', 'O113', 'O114', 'O115', 'O119', 'O161', 'O162', 'O163', 'O164', 'O165', 'O169' |
| **Hypertension, uncomplicated** | 'I10', 'I160', 'I169', 'I1A0', 'O10011', 'O10012', 'O10013', 'O10019', 'O1002', 'O1003' |
| **Mild liver disease** | 'A5145', 'A5274', 'B180', 'B181', 'B182', 'B188', 'B189', 'B1910', 'B1920', 'B199', 'B251', 'B581', 'K700', 'K7010', 'K7011', 'K702', 'K7030', 'K7031', 'K709', 'K713', 'K714', 'K7150', 'K7151', 'K716', 'K717', 'K718', 'K730', 'K731', 'K732', 'K738', 'K739', 'K740', 'K7400', 'K7401', 'K7402', 'K741', 'K742', 'K743', 'K744', 'K745', 'K7460', 'K7469', 'K751', 'K752', 'K753', 'K754', 'K7581', 'K7589', 'K759', 'K760', 'K761', 'K762', 'K763', 'K764', 'K7681', 'K7682', 'K7689', 'K769', 'K77' |
| **Severe liver disease** | 'B190', 'B1911', 'B1921', 'I8500', 'I8501', 'I8510', 'I8511', 'I864', 'K7040', 'K7041', 'K7210', 'K7211', 'K7290', 'K7291', 'K765', 'K766', 'K767', 'K9182', 'Z944' |
| **Chronic pulmonary disease** | 'J410', 'J411', 'J418', 'J42', 'J430', 'J431', 'J432', 'J438', 'J439', 'J440', 'J441', 'J4481', 'J4489', 'J449', 'J4520', 'J4521', 'J4522', 'J4530', 'J4531', 'J4532', 'J4540', 'J4541', 'J4542', 'J4550', 'J4551', 'J4552', 'J45901', 'J45902', 'J45909', 'J45990', 'J45991', 'J45998', 'J470', 'J471', 'J479', 'J4A0', 'J4A8', 'J4A9', 'J60', 'J61', 'J620', 'J628', 'J630', 'J631', 'J632', 'J633', 'J634', 'J635', 'J636', 'J64', 'J65', 'J660', 'J661', 'J662', 'J668', 'J670', 'J671', 'J672', 'J673', 'J674', 'J675', 'J676', 'J677', 'J678', 'J679', 'J684', 'J701', 'J703' |
| **Movement disorders (neurological)** | 'G08', 'G10', 'G110', 'G111', 'G1110', 'G1111', 'G1119', 'G112', 'G113', 'G114', 'G115', 'G116', 'G118', 'G119', 'G120', 'G121', 'G1220', 'G1221', 'G1222', 'G1223', 'G1224', 'G1225', 'G1229', 'G128', 'G129', 'G130', 'G131', 'G132', 'G138', 'G20', 'G20A1', 'G20A2', 'G20B1', 'G20B2', 'G20C', 'G210', 'G2111', 'G2119', 'G212', 'G213', 'G214', 'G218', 'G219', 'G230', 'G231', 'G232', 'G233', 'G238', 'G239', 'G2409', 'G241', 'G242', 'G248', 'G254', 'G255', 'G2570', 'G2571', 'G2579', 'G2581', 'G2582', 'G2583', 'G2589', 'G259', 'G26', 'G320', 'G3281', 'G3289', 'G803' |
| **Other neurological disorders** | 'E7500', 'E7501', 'E7502', 'E7509', 'E7510', 'E7511', 'E7519', 'E7523', 'E7525', 'E7526', 'E7527', 'E7528', 'E7529', 'E754', 'F05', 'F842', 'G35', 'G360', 'G368', 'G369', 'G370', 'G371', 'G372', 'G373', 'G374', 'G375', 'G378', 'G3781', 'G3789', 'G379', 'G47411', 'G47419', 'G47421', 'G47429', 'G890', 'G910', 'G911', 'G912', 'G913', 'G914', 'G918', 'G919', 'G930', 'G9340', 'G9341', 'G9342', 'G9343', 'G9344', 'G9349', 'G935', 'G936', 'G937', 'G9381', 'G9382', 'G9389', 'G939', 'G94', 'K7682', 'O99350', 'O99351', 'O99352', 'O99353', 'O99354', 'O99355', 'P9160', 'P9161', 'P9162', 'P9163' |
| **Seizure disorders** | 'G40001', 'G40009', 'G40011', 'G40019', 'G40101', 'G40109', 'G40111', 'G40119', 'G40201', 'G40209', 'G40211', 'G40219', 'G40301', 'G40309', 'G40311', 'G40319', 'G40401', 'G40409', 'G40411', 'G40419', 'G4042', 'G40501', 'G40509', 'G40801', 'G40802', 'G40803', 'G40804', 'G40811', 'G40812', 'G40813', 'G40814', 'G40821', 'G40822', 'G40823', 'G40824', 'G40833', 'G40834', 'G4089', 'G40901', 'G40909', 'G40911', 'G40919', 'G40A01', 'G40A09', 'G40A11', 'G40A19', 'G40B01', 'G40B09', 'G40B11', 'G40B19', 'G40C01', 'G40C09', 'G40C11', 'G40C19', 'R561', 'R569' |
| **Obesity** | 'E6601', 'E6609', 'E661', 'E662', 'E668', 'E669', 'O99210', 'O99211', 'O99212', 'O99213', 'O99214', 'O99215', 'R939', 'Z6830', 'Z6831', 'Z6832', 'Z6833', 'Z6834', 'Z6835', 'Z6836', 'Z6837', 'Z6838', 'Z6839', 'Z6841', 'Z6842', 'Z6843', 'Z6844', 'Z6845', 'Z6854' |
| **Paralysis** | 'G041', 'G800', 'G801', 'G802', 'G808', 'G809', 'G8100', 'G8101', 'G8102', 'G8103', 'G8104', 'G8110', 'G8111', 'G8112', 'G8113', 'G8114', 'G8190', 'G8191', 'G8192', 'G8193', 'G8194', 'G8220', 'G8221', 'G8222', 'G8250', 'G8251', 'G8252', 'G8253', 'G8254', 'G830', 'G8310', 'G8311', 'G8312', 'G8313', 'G8314', 'G8320', 'G8321', 'G8322', 'G8323', 'G8324', 'G8330', 'G8331', 'G8332', 'G8333', 'G8334', 'G834', 'G835', 'G8381', 'G8382', 'G8383', 'G8384', 'G8389', 'G839', 'I69031', 'I69032', 'I69033', 'I69034', 'I69039', 'I69041', 'I69042', 'I69043', 'I69044', 'I69049', 'I69051', 'I69052', 'I69053', 'I69054', 'I69059', 'I69061', 'I69062', 'I69063', 'I69064', 'I69065', 'I69069', 'I69131', 'I69132', 'I69133', 'I69134', 'I69139', 'I69141', 'I69142', 'I69143', 'I69144', 'I69149', 'I69151', 'I69152', 'I69153', 'I69154', 'I69159', 'I69161', 'I69162', 'I69163', 'I69164', 'I69165', 'I69169', 'I69231', 'I69232', 'I69233', 'I69234', 'I69239', 'I69241', 'I69242', 'I69243', 'I69244', 'I69249', 'I69251', 'I69252', 'I69253', 'I69254', 'I69259', 'I69261', 'I69262', 'I69263', 'I69264', 'I69265', 'I69269', 'I69331', 'I69332', 'I69333', 'I69334', 'I69339', 'I69341', 'I69342', 'I69343', 'I69344', 'I69349', 'I69351', 'I69352', 'I69353', 'I69354', 'I69359', 'I69361', 'I69362', 'I69363', 'I69364', 'I69365', 'I69369', 'I69831', 'I69832', 'I69833', 'I69834', 'I69839', 'I69841', 'I69842', 'I69843', 'I69844', 'I69849', 'I69851', 'I69852', 'I69853', 'I69854', 'I69859', 'I69861', 'I69862', 'I69863', 'I69864', 'I69865', 'I69869', 'I69931', 'I69932', 'I69933', 'I69934', 'I69939', 'I69941', 'I69942', 'I69943', 'I69944', 'I69949', 'I69951', 'I69952', 'I69953', 'I69954', 'I69959', 'I69961', 'I69962', 'I69963', 'I69964', 'I69965', 'I69969', 'R532' |
| **Peripheral vascular disorders** | 'A5200', 'A5201', 'A5202', 'A5209', 'I700', 'I701', 'I70201', 'I70202', 'I70203', 'I70208', 'I70209', 'I70211', 'I70212', 'I70213', 'I70218', 'I70219', 'I70221', 'I70222', 'I70223', 'I70228', 'I70229', 'I70231', 'I70232', 'I70233', 'I70234', 'I70235', 'I70238', 'I70239', 'I70241', 'I70242', 'I70243', 'I70244', 'I70245', 'I70248', 'I70249', 'I7025', 'I70261', 'I70262', 'I70263', 'I70268', 'I70269', 'I70291', 'I70292', 'I70293', 'I70298', 'I70299', 'I70301', 'I70302', 'I70303', 'I70308', 'I70309', 'I70311', 'I70312', 'I70313', 'I70318', 'I70319', 'I70321', 'I70322', 'I70323', 'I70328', 'I70329', 'I70331', 'I70332', 'I70333', 'I70334', 'I70335', 'I70338', 'I70339', 'I70341', 'I70342', 'I70343', 'I70344', 'I70345', 'I70348', 'I70349', 'I7035', 'I70361', 'I70362', 'I70363', 'I70368', 'I70369', 'I70391', 'I70392', 'I70393', 'I70398', 'I70399', 'I70401', 'I70402', 'I70403', 'I70408', 'I70409', 'I70411', 'I70412', 'I70413', 'I70418', 'I70419', 'I70421', 'I70422', 'I70423', 'I70428', 'I70429', 'I70431', 'I70432', 'I70433', 'I70434', 'I70435', 'I70438', 'I70439', 'I70441', 'I70442', 'I70443', 'I70444', 'I70445', 'I70448', 'I70449', 'I7045', 'I70461', 'I70462', 'I70463', 'I70468', 'I70469', 'I70491', 'I70492', 'I70493', 'I70498', 'I70499', 'I70501', 'I70502', 'I70503', 'I70508', 'I70509', 'I70511', 'I70512', 'I70513', 'I70518', 'I70519', 'I70521', 'I70522', 'I70523', 'I70528', 'I70529', 'I70531', 'I70532', 'I70533', 'I70534', 'I70535', 'I70538', 'I70539', 'I70541', 'I70542', 'I70543', 'I70544', 'I70545', 'I70548', 'I70549', 'I7055', 'I70561', 'I70562', 'I70563', 'I70568', 'I70569', 'I70591', 'I70592', 'I70593', 'I70598', 'I70599', 'I70601', 'I70602', 'I70603', 'I70608', 'I70609', 'I70611', 'I70612', 'I70613', 'I70618', 'I70619', 'I70621', 'I70622', 'I70623', 'I70628', 'I70629', 'I70631', 'I70632', 'I70633', 'I70634', 'I70635', 'I70638', 'I70639', 'I70641', 'I70642', 'I70643', 'I70644', 'I70645', 'I70648', 'I70649', 'I7065', 'I70661', 'I70662', 'I70663', 'I70668', 'I70669', 'I70691', 'I70692', 'I70693', 'I70698', 'I70699', 'I70701', 'I70702', 'I70703', 'I70708', 'I70709', 'I70711', 'I70712', 'I70713', 'I70718', 'I70719', 'I70721', 'I70722', 'I70723', 'I70728', 'I70729', 'I70731', 'I70732', 'I70733', 'I70734', 'I70735', 'I70738', 'I70739', 'I70741', 'I70742', 'I70743', 'I70744', 'I70745', 'I70748', 'I70749', 'I7075', 'I70761', 'I70762', 'I70763', 'I70768', 'I70769', 'I70791', 'I70792', 'I70793', 'I70798', 'I70799', 'I708', 'I7090', 'I7091', 'I7092', 'I7100', 'I7101', 'I71010', 'I71011', 'I71012', 'I71019', 'I7102', 'I7103', 'I711', 'I7110', 'I7111', 'I7112', 'I7113', 'I712', 'I7120', 'I7121', 'I7122', 'I7123', 'I713', 'I7130', 'I7131', 'I7132', 'I7133', 'I714', 'I7140', 'I7141', 'I7142', 'I7143', 'I715', 'I7150', 'I7151', 'I7152', 'I716', 'I7160', 'I7161', 'I7162', 'I718', 'I719', 'I720', 'I721', 'I722', 'I723', 'I724', 'I725', 'I726', 'I728', 'I729', 'I7301', 'I731', 'I7381', 'I7389', 'I739', 'I7401', 'I7409', 'I7410', 'I7411', 'I7419', 'I742', 'I743', 'I744', 'I745', 'I748', 'I749', 'I75011', 'I75012', 'I75013', 'I75019', 'I75021', 'I75022', 'I75023', 'I75029', 'I7581', 'I7589', 'I770', 'I771', 'I772', 'I773', 'I774', 'I775', 'I776', 'I7770', 'I7771', 'I7772', 'I7773', 'I7774', 'I7775', 'I7776', 'I7777', 'I7779', 'I77810', 'I77811', 'I77812', 'I77819', 'I7782', 'I7789', 'I779', 'I780', 'I781', 'I788', 'I789', 'I790', 'I791', 'I798', 'K551', 'Z95820', 'Z95828' |
| **Psychoses** | 'F060', 'F061', 'F062', 'F0630', 'F0633', 'F11150', 'F11151', 'F11159', 'F11250', 'F11251', 'F11259', 'F11950', 'F11951', 'F11959', 'F12150', 'F12151', 'F12159', 'F12250', 'F12251', 'F12259', 'F12950', 'F12951', 'F12959', 'F13150', 'F13151', 'F13159', 'F13250', 'F13251', 'F13259', 'F13950', 'F13951', 'F13959', 'F14150', 'F14151', 'F14159', 'F14250', 'F14251', 'F14259', 'F14950', 'F14951', 'F14959', 'F15150', 'F15151', 'F15159', 'F15250', 'F15251', 'F15259', 'F15950', 'F15951', 'F15959', 'F16150', 'F16151', 'F16159', 'F16250', 'F16251', 'F16259', 'F16950', 'F16951', 'F16959', 'F18150', 'F18151', 'F18159', 'F18250', 'F18251', 'F18259', 'F18950', 'F18951', 'F18959', 'F19150', 'F19151', 'F19159', 'F19250', 'F19251', 'F19259', 'F19950', 'F19951', 'F19959', 'F200', 'F201', 'F202', 'F203', 'F205', 'F2081', 'F2089', 'F209', 'F21', 'F22', 'F23', 'F24', 'F250', 'F251', 'F258', 'F259', 'F28', 'F29', 'F3010', 'F3011', 'F3012', 'F3013', 'F302', 'F303', 'F304', 'F308', 'F309', 'F310', 'F3110', 'F3111', 'F3112', 'F3113', 'F312', 'F3130', 'F3131', 'F3132', 'F314', 'F315', 'F3160', 'F3161', 'F3162', 'F3163', 'F3164', 'F3170', 'F3171', 'F3172', 'F3173', 'F3174', 'F3175', 'F3176', 'F3177', 'F3178', 'F3181', 'F3189', 'F319', 'F324', 'F325', 'F3340', 'F3341', 'F3342', 'F340', 'F348', 'F3481', 'F3489', 'F349', 'F39', 'F4489', 'F843' |
| **Pulmonary circulation disorders** | 'I270', 'I271', 'I272', 'I2720', 'I2721', 'I2722', 'I2723', 'I2724', 'I2729', 'I2781', 'I2782', 'I2783', 'I2789', 'I279', 'I280', 'I281', 'I288', 'I289' |
| **Moderate renal failure** | 'N183', 'N1830', 'N1831', 'N1832', 'N189', 'N19' |
| **Severe renal failure (end-stage)** | 'I120', 'I1311', 'I132', 'N184', 'N185', 'N186', 'Z4901', 'Z4902', 'Z4931', 'Z4932', 'Z9115', 'Z91151', 'Z91158', 'Z940', 'Z992' |
| **Hypothyroidism** | 'E000', 'E001', 'E002', 'E009', 'E010', 'E011', 'E012', 'E018', 'E02', 'E030', 'E031', 'E032', 'E033', 'E034', 'E035', 'E038', 'E039', 'E890' |
| **Peptic ulcer disease** | 'K250', 'K251', 'K252', 'K253', 'K254', 'K255', 'K256', 'K257', 'K259', 'K260', 'K261', 'K262', 'K263', 'K264', 'K265', 'K266', 'K267', 'K269', 'K270', 'K271', 'K272', 'K273', 'K274', 'K275', 'K276', 'K277', 'K279', 'K280', 'K281', 'K282', 'K283', 'K284', 'K285', 'K286', 'K287', 'K289' |
| **Valvular disease** | 'A1884', 'A3282', 'A3951', 'A5203', 'B3321', 'B376', 'I011', 'I018', 'I019', 'I020', 'I050', 'I051', 'I052', 'I058', 'I059', 'I060', 'I061', 'I062', 'I068', 'I069', 'I070', 'I071', 'I072', 'I078', 'I079', 'I080', 'I081', 'I082', 'I083', 'I088', 'I089', 'I091', 'I0989', 'I330', 'I339', 'I340', 'I341', 'I342', 'I348', 'I3481', 'I3489', 'I349', 'I350', 'I351', 'I352', 'I358', 'I359', 'I360', 'I361', 'I362', 'I368', 'I369', 'I370', 'I371', 'I372', 'I378', 'I379', 'I38', 'I39', 'M3211', 'Q220', 'Q221', 'Q222', 'Q223', 'Q224', 'Q225', 'Q226', 'Q228', 'Q229', 'Q230', 'Q231', 'Q232', 'Q233', 'Q234', 'Q238', 'Q239', 'T8201XA', 'T8201XD', 'T8201XS', 'T8202XA', 'T8202XD', 'T8202XS', 'T8203XA', 'T8203XD', 'T8203XS', 'T8209XA', 'T8209XD', 'T8209XS', 'T82221A', 'T82221D', 'T82221S', 'T82222A', 'T82222D', 'T82222S', 'T82223A', 'T82223D', 'T82223S', 'T82228A', 'T82228D', 'T82228S', 'T826XXA', 'T826XXD', 'T826XXS', 'Z952', 'Z953', 'Z954' |
| **Weight loss (cachexia)** | 'E40', 'E41', 'E42', 'E43', 'E440', 'E441', 'E45', 'E46', 'E640', 'E88A', 'O2510', 'O2511', 'O2512', 'O2513', 'O252', 'O253', 'R634', 'R64' |

| **Clinical Outcomes** | |
| --- | --- |
| **Cardiogenic Shock** | **R57.0** - Cardiogenic shock |
| **Cardiac Arrythmias (Atrial Fibrillation and Ventricular Tachycardia/Fibrillation)** | **I48.0** - Paroxysmal atrial fibrillation **I48.1** - Persistent atrial fibrillation **I48.2** - Chronic atrial fibrillation **I48.91** - Unspecified atrial fibrillation  **I49.01** - Ventricular fibrillation **I49.02** - Ventricular flutter  **I47.2** - Ventricular tachycardia |
| **Atrial Fibrillation** | **I48.0** - Paroxysmal atrial fibrillation **I48.1** - Persistent atrial fibrillation **I48.2** - Chronic atrial fibrillation **I48.91** - Unspecified atrial fibrillation |
| **Ventricular Tachycardia/Fibrillation** | **I49.01** - Ventricular fibrillation **I49.02** - Ventricular flutter  **I47.2** - Ventricular tachycardia |
| **Cardiac Arrest** | **I46.2** - Cardiac arrest due to underlying cardiac condition **I46.8** - Cardiac arrest due to other underlying condition **I46.9** - Cardiac arrest, cause unspecified |
| **Heart Failure (HF)** | **I50.1** - Left ventricular failure **I50.20** - Unspecified systolic (congestive) heart failure **I50.21** - Acute systolic (congestive) heart failure **I50.22** - Chronic systolic (congestive) heart failure **I50.23** - Acute on chronic systolic (congestive) heart failure **I50.30** - Unspecified diastolic (congestive) heart failure **I50.31** - Acute diastolic (congestive) heart failure **I50.32** - Chronic diastolic (congestive) heart failure **I50.33** - Acute on chronic diastolic (congestive) heart failure **I50.40** - Unspecified combined systolic and diastolic heart failure **I50.41** - Acute combined systolic and diastolic heart failure **I50.42** - Chronic combined systolic and diastolic heart failure **I50.43** - Acute on chronic combined systolic and diastolic heart failure **I50.9** - Heart failure, unspecified |
| **Myocardial Infarction (MI)** | **I21.0** - ST elevation (STEMI) myocardial infarction of anterior wall **I21.1** - STEMI myocardial infarction of inferior wall **I21.2** - STEMI myocardial infarction of other sites **I21.3** - ST elevation (STEMI) myocardial infarction of unspecified site **I21.4** - Non-ST elevation (NSTEMI) myocardial infarction **I21.A1** - Myocardial infarction type 2 **I21.A9** - Other myocardial infarction type **I21.9** - Acute myocardial infarction, unspecified **I22.0** - Subsequent STEMI of anterior wall **I22.1** - Subsequent STEMI of inferior wall **I22.2** - Subsequent STEMI of other sites **I22.8** - Subsequent STEMI of unspecified site **I22.9** - Subsequent NSTEMI myocardial infarction |
| **Venous Thromboembolism (VTE) (Pulmonary Embolism (PE) and Deep Vein Thrombosis (DVT))** | **I26.01** - Septic pulmonary embolism with acute cor pulmonale **I26.02** - Saddle embolus of pulmonary artery with acute cor pulmonale **I26.09** - Other pulmonary embolism with acute cor pulmonale **I26.90** - Septic pulmonary embolism without acute cor pulmonale **I26.92** - Saddle embolus of pulmonary artery without acute cor pulmonale  **I26.99** - Other pulmonary embolism without acute cor pulmonale  **I82.401** - Acute embolism and thrombosis of unspecified deep veins of right lower extremity **I82.402** - Acute embolism and thrombosis of unspecified deep veins of left lower extremity **I82.403** - Acute embolism and thrombosis of unspecified deep veins of lower extremity, bilateral **I82.409** - Acute embolism and thrombosis of unspecified deep veins of unspecified lower extremity **I82.411** - Acute embolism and thrombosis of right femoral vein **I82.412** - Acute embolism and thrombosis of left femoral vein **I82.413** - Acute embolism and thrombosis of femoral vein, bilateral **I82.419** - Acute embolism and thrombosis of femoral vein, unspecified **I82.421** - Acute embolism and thrombosis of right popliteal vein **I82.422** - Acute embolism and thrombosis of left popliteal vein **I82.423** - Acute embolism and thrombosis of popliteal vein, bilateral **I82.429** - Acute embolism and thrombosis of popliteal vein, unspecified **I82.431** - Acute embolism and thrombosis of right tibial vein **I82.432** - Acute embolism and thrombosis of left tibial vein **I82.433** - Acute embolism and thrombosis of tibial vein, bilateral **I82.439** - Acute embolism and thrombosis of tibial vein, unspecified **I82.441** - Acute embolism and thrombosis of right peroneal vein **I82.442** - Acute embolism and thrombosis of left peroneal vein **I82.443** - Acute embolism and thrombosis of peroneal vein, bilateral **I82.449** - Acute embolism and thrombosis of peroneal vein, unspecified **I82.4Y1** - Acute embolism and thrombosis of other specified deep vein of right lower extremity **I82.4Y2** - Acute embolism and thrombosis of other specified deep vein of left lower extremity **I82.4Y3** - Acute embolism and thrombosis of other specified deep vein of lower extremity, bilateral **I82.4Y9** - Acute embolism and thrombosis of other specified deep vein of unspecified lower extremity  **I82.4Z1 -**Acute embolism and thrombosis of unspecified deep veins of right distal lower extremity  **I82.4Z2 -**Acute embolism and thrombosis of unspecified deep veins of left distal lower extremity  **I82.4Z3**…… bilateral  **I82.4Z9 -**Acute embolism and thrombosis of unspecified deep veins of unspecified distal lower extremity  **I26.99** - Other pulmonary embolism without acute cor pulmonale |
| **Pulmonary Embolism (PE)** | **I26.01** - Septic pulmonary embolism with acute cor pulmonale **I26.02** - Saddle embolus of pulmonary artery with acute cor pulmonale **I26.09** - Other pulmonary embolism with acute cor pulmonale **I26.90** - Septic pulmonary embolism without acute cor pulmonale **I26.92** - Saddle embolus of pulmonary artery without acute cor pulmonale **I26.99** - Other pulmonary embolism without acute cor pulmonale |
| **Deep Vein Thrombosis (DVT)** | **I82.401** - Acute embolism and thrombosis of unspecified deep veins of right lower extremity **I82.402** - Acute embolism and thrombosis of unspecified deep veins of left lower extremity **I82.403** - Acute embolism and thrombosis of unspecified deep veins of lower extremity, bilateral **I82.409** - Acute embolism and thrombosis of unspecified deep veins of unspecified lower extremity **I82.411** - Acute embolism and thrombosis of right femoral vein **I82.412** - Acute embolism and thrombosis of left femoral vein **I82.413** - Acute embolism and thrombosis of femoral vein, bilateral **I82.419** - Acute embolism and thrombosis of femoral vein, unspecified **I82.421** - Acute embolism and thrombosis of right popliteal vein **I82.422** - Acute embolism and thrombosis of left popliteal vein **I82.423** - Acute embolism and thrombosis of popliteal vein, bilateral **I82.429** - Acute embolism and thrombosis of popliteal vein, unspecified **I82.431** - Acute embolism and thrombosis of right tibial vein **I82.432** - Acute embolism and thrombosis of left tibial vein **I82.433** - Acute embolism and thrombosis of tibial vein, bilateral **I82.439** - Acute embolism and thrombosis of tibial vein, unspecified **I82.441** - Acute embolism and thrombosis of right peroneal vein **I82.442** - Acute embolism and thrombosis of left peroneal vein **I82.443** - Acute embolism and thrombosis of peroneal vein, bilateral **I82.449** - Acute embolism and thrombosis of peroneal vein, unspecified **I82.4Y1** - Acute embolism and thrombosis of other specified deep vein of right lower extremity **I82.4Y2** - Acute embolism and thrombosis of other specified deep vein of left lower extremity **I82.4Y3** - Acute embolism and thrombosis of other specified deep vein of lower extremity, bilateral **I82.4Y9** - Acute embolism and thrombosis of other specified deep vein of unspecified lower extremity  **I82.4Z1 -**Acute embolism and thrombosis of unspecified deep veins of right distal lower extremity  **I82.4Z2 -**Acute embolism and thrombosis of unspecified deep veins of left distal lower extremity  **I82.4Z3**…… bilateral  **I82.4Z9 -**Acute embolism and thrombosis of unspecified deep veins of unspecified distal lower extremity |
| **Transient Ischemic Attack (TIA)** | **G45.0** - Vertebro-basilar artery syndrome **G45.1** - Carotid artery syndrome (hemispheric) **G45.2** - Multiple and bilateral precerebral artery syndromes **G45.8** - Other transient cerebral ischemic attacks and related syndromes **G45.9** - Transient cerebral ischemic attack, unspecified |
| **Ischemic Cerebrovascular Accident (CVA)** | **I63.00** - Cerebral infarction due to thrombosis of unspecified precerebral artery **I63.011** - Cerebral infarction due to thrombosis of right vertebral artery **I63.012** - Cerebral infarction due to thrombosis of left vertebral artery **I63.013** - Cerebral infarction due to thrombosis of bilateral vertebral arteries **I63.019** - Cerebral infarction due to thrombosis of unspecified vertebral artery **I63.02** - Cerebral infarction due to thrombosis of basilar artery **I63.031** - Cerebral infarction due to thrombosis of right carotid artery **I63.032** - Cerebral infarction due to thrombosis of left carotid artery **I63.033** - Cerebral infarction due to thrombosis of bilateral carotid arteries **I63.039** - Cerebral infarction due to thrombosis of unspecified carotid artery **I63.09** - Cerebral infarction due to thrombosis of other precerebral arteries **I63.10** - Cerebral infarction due to embolism of unspecified precerebral artery **I63.111** - Cerebral infarction due to embolism of right vertebral artery **I63.112** - Cerebral infarction due to embolism of left vertebral artery **I63.113** - Cerebral infarction due to embolism of bilateral vertebral arteries **I63.119** - Cerebral infarction due to embolism of unspecified vertebral artery **I63.12** - Cerebral infarction due to embolism of basilar artery **I63.131** - Cerebral infarction due to embolism of right carotid artery **I63.132** - Cerebral infarction due to embolism of left carotid artery **I63.133** - Cerebral infarction due to embolism of bilateral carotid arteries **I63.139** - Cerebral infarction due to embolism of unspecified carotid artery **I63.19** - Cerebral infarction due to embolism of other precerebral arteries **I63.20** - Cerebral infarction due to unspecified occlusion or stenosis of unspecified precerebral artery **I63.30** - Cerebral infarction due to thrombosis of unspecified cerebral artery **I63.311** - Cerebral infarction due to thrombosis of right middle cerebral artery **I63.312** - Cerebral infarction due to thrombosis of left middle cerebral artery **I63.313** - Cerebral infarction due to thrombosis of bilateral middle cerebral arteries **I63.319** - Cerebral infarction due to thrombosis of unspecified middle cerebral artery **I63.321** - Cerebral infarction due to thrombosis of right anterior cerebral artery **I63.322** - Cerebral infarction due to thrombosis of left anterior cerebral artery **I63.323** - Cerebral infarction due to thrombosis of bilateral anterior cerebral arteries **I63.329** - Cerebral infarction due to thrombosis of unspecified anterior cerebral artery **I63.331** - Cerebral infarction due to thrombosis of right posterior cerebral artery **I63.332** - Cerebral infarction due to thrombosis of left posterior cerebral artery **I63.333** - Cerebral infarction due to thrombosis of bilateral posterior cerebral arteries **I63.339** - Cerebral infarction due to thrombosis of unspecified posterior cerebral artery **I63.341** - Cerebral infarction due to thrombosis of right cerebellar artery **I63.342** - Cerebral infarction due to thrombosis of left cerebellar artery **I63.343** - Cerebral infarction due to thrombosis of bilateral cerebellar arteries **I63.349** - Cerebral infarction due to thrombosis of unspecified cerebellar artery **I63.39** - Cerebral infarction due to thrombosis of other cerebral arteries **I63.40** - Cerebral infarction due to embolism of unspecified cerebral artery **I63.50** - Cerebral infarction due to unspecified occlusion or stenosis of unspecified cerebral artery **I63.6** - Cerebral infarction due to cerebral venous thrombosis, nonpyogenic **I63.8** - Other cerebral infarction **I63.9** - Cerebral infarction, unspecified |

**S2 Table:**

Strengthening the Reporting of Observational Studies in Epidemiology (STROBE) guidelines to report the study findings.

STROBE Statement—Checklist of items that should be included in reports of ***cross-sectional studies***

|  | **Item No** | **Recommendation** | **Page No** |
| --- | --- | --- | --- |
| **Title and abstract** | 1 | (*a*) Indicate the study’s design with a commonly used term in the title or the abstract | 1 |
|  |  | (*b*) Provide in the abstract an informative and balanced summary of what was done and what was found | 2 |
| **Introduction** | | | |
| Background/rationale | 2 | Explain the scientific background and rationale for the investigation being reported | 4 |
| Objectives | 3 | State specific objectives, including any prespecified hypotheses | 4 |
| **Methods** | | | |
| Study design | 4 | Present key elements of study design early in the paper | 4,5 |
| Setting | 5 | Describe the setting, locations, and relevant dates, including periods of recruitment, exposure, follow-up, and data collection | 4,5 |
| Participants | 6 | (*a*) Give the eligibility criteria, and the sources and methods of selection of participants | 5 |
| Variables | 7 | Clearly define all outcomes, exposures, predictors, potential confounders, and effect modifiers. Give diagnostic criteria, if applicable | 4,5,6,7 |
| Data sources/ measurement | 8* | For each variable of interest, give sources of data and details of methods of assessment (measurement). Describe comparability of assessment methods if there is more than one group | 4,5,6,7 |
| Bias | 9 | Describe any efforts to address potential sources of bias | 7 |
| Study size | 10 | Explain how the study size was arrived at | 4,5,6,7 |
| Quantitative variables | 11 | Explain how quantitative variables were handled in the analyses. If applicable, describe which groupings were chosen and why | 4,5,6,7 |
| Statistical methods | 12 | (*a*) Describe all statistical methods, including those used to control for confounding | 7 |
|  |  | (*b*) Describe any methods used to examine subgroups and interactions | 7 |
|  |  | (*c*) Explain how missing data were addressed | 5 |
|  |  | (*d*) If applicable, describe analytical methods taking account of sampling strategy | 4,5,6,7 |
|  |  | (*e*) Describe any sensitivity analyses | N/A |
| **Results** | | | |
| Participants | 13* | (a) Report numbers of individuals at each stage of study—eg numbers potentially eligible, examined for eligibility, confirmed eligible, included in the study, completing follow-up, and analysed | 7 |
|  |  | (b) Give reasons for non-participation at each stage | 7 |
|  |  | (c) Consider use of a flow diagram | Figure 1 |
| Descriptive data | 14* | (a) Give characteristics of study participants (eg demographic, clinical, social) and information on exposures and potential confounders | 7,8 |
|  |  | (b) Indicate number of participants with missing data for each variable of interest | 7 |
| Outcome data | 15* | Report numbers of outcome events or summary measures | 7,8,9,10,11 |
| Main results | 16 | (*a*) Give unadjusted estimates and, if applicable, confounder-adjusted estimates and their precision (eg, 95% confidence interval). Make clear which confounders were adjusted for and why they were included | 7,8,9,10,11, Figure 2, Figure 3 |
|  |  | (*b*) Report category boundaries when continuous variables were categorized | 7,8,9,10,11 |
|  |  | (*c*) If relevant, consider translating estimates of relative risk into absolute risk for a meaningful time period | N/A |
| Other analyses | 17 | Report other analyses done—eg analyses of subgroups and interactions, and sensitivity analyses | N/A |
| **Discussion** | | | |
| Key results | 18 | Summarise key results with reference to study objectives | 11,12,13,14,15 |
| Limitations | 19 | Discuss limitations of the study, taking into account sources of potential bias or imprecision. Discuss both direction and magnitude of any potential bias | 14,15 |
| Interpretation | 20 | Give a cautious overall interpretation of results considering objectives, limitations, multiplicity of analyses, results from similar studies, and other relevant evidence | 11,12,13,14,15 |
| Generalisability | 21 | Discuss the generalisability (external validity) of the study results | 14,15 |
| **Other information** | | | |
| Funding | 22 | Give the source of funding and the role of the funders for the present study and, if applicable, for the original study on which the present article is based | 1 |

*Give information separately for exposed and unexposed groups.

**Note:** An Explanation and Elaboration article discusses each checklist item and gives methodological background and published examples of transparent reporting. The STROBE checklist is best used in conjunction with this article (freely available on the Web sites of PLoS Medicine at http://www.plosmedicine.org/, Annals of Internal Medicine at http://www.annals.org/, and Epidemiology at http://www.epidem.com/). Information on the STROBE Initiative is available at www.strobe-statement.org.
